# Natural molecule potentiates colistin efficacy *in vivo* via modulating adaptive LPS modifications and ferroptotic-like damages

**DOI:** 10.1080/21505594.2026.2707759

**Published:** 2026-07-21

**Authors:** Hui-Hui Zhang, Yi-Dan Cao, Ying-Ying Xie, Yu-Ze Li, Xiao-Na Fan, Qiu-Yue Diao, Yu-Jiao Liang, Li-Ren He, Zi-Xing Zhong, Li-Juan Xia, Ze-Hua Cui, Xiao-Ping Liao, Xin-Lei Lian, Dong-Hao Zhao, Jian Sun, Hao Ren

**Affiliations:** a State Key Laboratory of Animal Disease Control and Prevention, College of Veterinary Medicine, South China Agricultural University, Guangzhou, China; b National Risk Assessment Laboratory for Antimicrobial Resistance of Animal Original Bacteria, South China Agricultural University, Guangzhou, China; c Guangdong Provincial Key Laboratory of Veterinary Pharmaceutics Development and Safety Evaluation, South China Agricultural University, Guangzhou, China

**Keywords:** Antibiotic resistance, colistin, LPS modifications, two-component system, ferroptotic-like damage

## Abstract

Colistin holds clinical importance as a last resort therapy against infections caused by multidrug-resistant Gram-negative pathogens. In addition to well-studied plasmid-encoded *mcr* alleles, the adaptive mechanisms primed by host conditions are increasingly evidenced to be responsible for suboptimal efficacy of colistin in clinical practice, yet have long been neglected. Herein, we reported the identification of baicalein that potentiates colistin killing *in vitro* and *in vivo* by manipulating bacterial labile iron pool, where ferrous iron accumulates at the expense of ferric and total iron. This change in iron abundance leads to inactivation of the PmrA/B two-component system and subsequent PmrA/B-dependent lipopolysaccharide modifications, finally promoting colistin to bind and destabilize the bacterial membrane. Meanwhile, increased ferrous iron, in response to colistin-induced ROS, results in ferroptotic-like damage, which generates lethal reactive electrophilic species to deteriorate the essential cellular constituents. These data underscore an exploitable link between iron homeostasis and colistin susceptibility, as both primary and secondary actions of colistin respond to the shifts in cellular labile iron. To sum, this study offers a translationally viable regimen of baicalein-colistin combination against Gram-negative pathogen infection and reveals the cellular labile iron as a generally applicable target for developing next-generation colistin adjuvants.

## Introduction

Introducing antibiotics to clinical practice considerably reshaped modern medicine, converting once-deadly bacterial infections under control [1]. However, cumulative and indiscriminate use of antibiotics over decades creates another looming threat of the current antimicrobial resistance (AMR) crisis [2]. To address the gradually decreasing efficacy of antibiotics in use, a plethora of novel strategies have been included to fill the void in the unmet demands of new regimens. As such, identifying antimicrobial compounds that exploit new targets and modes of action receives immense attention, especially those targeting Gram-negative pathogens that are designated as a critical threat by the World Health Organization [3]. For example, the newly developed iChip enables the discovery of several new antibiotics from the microbial “dark matter” of unculturable bacteria. This encouraging technique yields the new solutions based on teixobactin and clovibactin, which bind to the peptidoglycan precursor lipid II for cell‑wall disruptions [4–6]. Besides panning antibiotics by chemical biology strategies, growing artificial intelligence (AI) approaches are applied to rapidly offer the new antibacterial scaffolds, where the new molecule halicin was generated active against a wide panel of pathogens [7]. These exceptional efforts have profoundly expanded the arsenal to combat bacterial infections and sidestep the current resistant determinants. However, to advance as translational candidates, these promising leads still warrant time and money-consuming progress to clearly elucidate the potential toxicities, side effects, as well as the pharmacodynamic behaviors [8], portending that the number of viable drugs licensed for clinics still lag behind the pace of AMR dissemination.

In comparison to the introduction of novel antimicrobials, the combination therapy based on antibiotic adjuvants to enhance the potency, spectrum or performance of existing drugs possesses special advantages in accessibility, cost, and time consumption [9]. For instance, the β-lactamase inhibitors like clavulanic acid with cephalosporins have been one of the most successful combinations that are widely prescribed in clinical practices to date [10]. By combination with appropriate adjuvants, advantages are confirmed in restoring antibiotic sensitivity, refining the dose regimens, and obstructing the emergence of resistance, rendering the existing well-approved antibiotics revitalized into a treatment choice. Particularly, as an antibiotic of last resort, colistin (CS) has reemerged in the clinic for the treatment of carbapenem-resistant Gram-negative bacteria, but its utility is threatened by the surging prevalence of CS resistance [11]. This resistance, either mediated by chromosomal mutations or the mobilized CS resistance gene (*mcr*-1) and its variants on plasmids, is able to modulate chemical modifications in lipid A moieties of LPS, thereby reducing the CS binding and killing to a larger extent [12]. Given the limitations of CS in dose-dependent nephrotoxicity and neurotoxicity [13], CS in excess for overcoming resistance is not technically feasible, suggesting pairing with adjuvants in combination as a potential solution to balance the efficacy and safety. Thus far, several prior studies have provided promising candidates as the CS resistance breakers. For instance, a neurohormone, melatonin, has been found to restore the CS activity against *mcr*-positive pathogens via membrane disruption and oxidative stress [14]. Likewise, Carfrae and coworkers identified that the biotin biosynthesis inhibitor MAC13772 exclusively acts on the bacteria bearing *mcr*-1 to overcome the CS resistance [15]. However, it should be noted that, beyond aforementioned acquired resistance mechanisms, certain bacterial species (e.g. *Salmonella* spp., *E. coli* or *K. pneumoniae*, which can internalize into or be phagocytosed by the macrophage) are found more resistant to CS at host conditions by transiently activating several two component systems, even without genetic determinants of CS resistance [16,17]. In this regard, the resistance caused by this adaptive mechanism may be a driver behind the suboptimal responses in patients receiving a CS regimen at regular doses [18–20]. As supported by this, these adaptive resistances might be equally important to the acquired CS resistance, yet only a handful of previous studies have taken this into consideration in the development of CS adjuvants [21]. Recently, the application of host-mimicking medium like LPM (low-phosphate, low-magnesium medium) offers an opportunity to study bacterial response under such intracellular-like condition. Therefore, several studies attempted to unveil potential breakers that can overcome aforementioned adaptive resistance [22].

In this work, we identified baicalein (BAI) that potentiates CS activity in both host-mimicking medium and *in vivo*. We present the mechanistic studies showing that synergism between BAI and CS depends on its ability to manipulate the bacterial cellular labile iron, where the ferrous iron accumulates at the expense of ferric and total iron. This iron dysregulation promotes both the primary action of CS to bind the bacterial membrane and its secondary action to induce highly reactive radicals, which collectively dictate the enhanced activity of CS. The present work not only explores the potential of BAI-CS combination in escalating the emerging threat of hard-to-treat Gram-negative infections but also unravels a hidden nexus between cellular labile iron with CS susceptibility, which can be deemed as a generally applicable pathway for CS adjuvant development.

## Materials and methods

### Bacterial strains and chemicals

All strains used in this study were stored in 30% glycerol at −80°C until experiments and listed in Table S2. All *Salmonella* experiments were performed on *S*. Tm ATCC 14,028 or its homologous derivatives unless otherwise stated. The bacteria were routinely cultured in Luria-Bertani (LB) broth or agar medium. The subculture was diluted by 1:100 and grown in low-phosphate, low-magnesium medium [LPM, host-mimicking medium, composition: 5 mM KCl, 7.5 mM (NH4)_2_SO_4_, 0.5 mM K_2_SO_4_, 80 mM MES (pH 5.8), 0.1% casamino acids, 0.3% (v/v) glycerol, 24 μM MgCl_2_, and 337 μM PO_4_^3−^] [23]. All chemicals and reagents used in this study not specifically mentioned were of analytical grade and commercially available (from Macklin, Shanghai, China).

### Primary screening for the adaptive CS resistance breakers

Preliminary screening was performed on a total of 80 chemicals from the lab collection using the LPM. Briefly, each chemical was prepared at the indicated concentration in DMSO and aliquoted into 96-well microtiter plates (10 µL/ well). Each well was then supplemented with 190 µL of LPM medium containing 5 × 10^3^ bacterial cells and the appropriate concentration of CS (C114323, Aladdin, China), resulting in a final DMSO concentration of 2% (v/v). The cultures were incubated at 37°C for 16–18 h, after which the optical density (OD_600_) of each well was measured using a multi-functional microplate reader (PerkinElmer, USA). The candidate chemicals that potentiate CS were identified according to previously published protocols, in which the drug interaction was interpreted by the $\tilde{\varepsilon }$ value [21]. The $\tilde{\varepsilon }$ was value was calculated using the following equation: $\tilde{\varepsilon }$  = (*W*_XY_ − *W*_X_*W*_Y_)/|$\tilde{W}$_XY_ − *W*_X_*W*_Y_|, where $\tilde{W}$_XY_ = min [*W*_X_,*W*_Y_], if *W*_XY_ > *W*_X_*W*_Y_ and is 0 otherwise. The *W*_X_, *W*_Y_ and *W*_XY_ stand for normalized reduced growth rate caused by the drugs (*W*_X_ = *g*_X_/*g*_*φ*_, *W*_Y_ = *g*_Y_/*g*_*φ*_, *W*_XY_ = *g*_XY_/*g*_*φ*_), where *g*_X_, *g*_X_, *g*_XY_ and *g*φ represent the measured growth rates with single drug, the drug combination, and no drug treatment, respectively. If $\tilde{W}$_XY_ was numerically greater than min [W_X_, W_Y_], then $\tilde{\varepsilon }$ was equal to {(W_XY_ − min [W_X_, W_Y_])/(1 − min [W_X_, W_Y_]) + 1. An $\tilde{\varepsilon }$ value between −1 and −0.5 was interpreted as synergistic, whereas all other interactions were categorized as additive or antagonistic.

### Antimicrobial susceptibility testing and checkerboard assay

The antimicrobial susceptibility testing for CS was performed according to standard EUCAST guidelines with appropriate adjustments. In brief, the overnight bacterial culture was first re-suspended in LPM to ~1 × 10^8^ CFU mL^−1^ (~OD_600_ = 0.25), subsequently serially diluted to a final concentration of ~1 × 10^6^ CFU mL^−1^, and the actual bacterial cell density was further confirmed by colony enumeration on agar plates. The inoculum was then exposed to CS with or without candidate chemicals in 96-well microplates. After incubation at 37°C for 18 h, the minimum drug concentration that inhibited bacterial growth was defined as the MIC for evaluation.

To evaluate the synergistic effects between CS and the candidate chemicals, we employed the Synergyfinder 3.0 and the checkerboard assay. The specific method was as follows: two drugs were serially diluted two-fold, and 50 μL of each at different concentrations was combined in the 96-well plate, followed by the addition of 100 μL of bacterial suspension (1 × 10^6^ CFU mL^−1^). After incubation at 37°C for 18 h, the interaction between drug pairs was assessed by calculating the Bliss score (SynergyFinder 3.0) or fractional inhibitory concentration index (FICI). The FICI was calculated using the formula:

$$\begin{array}{c} \mathrm{FI}C_{\mathrm{Index}}=\frac{\mathrm{MI}C_{\mathrm{AB}}}{\mathrm{MI}C_{A}}+\frac{\mathrm{MI}C_{\mathrm{BA}}}{\mathrm{MI}C_{B}}=\mathrm{FI}C_{A}+\mathrm{FI}C_{B} \end{array}$$

The combined MIC values were defined as: MIC_AB_ (MIC of A with B) and MIC_BA_ (MIC of B with A). Individual MICs were denoted as MIC_A_ (A alone) and MIC_B_ (B alone). The FIC of compound A or B is defined as FIC_A_ and FIC_B_. A synergistic interaction was defined as FICI ≤ 0.5.

### Cell viability analysis

Cell viability was assessed using the Cell Counting Kit-8 assay (CCK-8, Vazyme, China). The cell suspension (500 μL well^−1^) was inoculated in 24-well plates. Pre-incubation was carried out in a humidified incubator for 16–24 h (37°C, 5% CO_2_). Then candidate chemical was supplemented and incubated for 12 h. According to the manufacturer’s instructions, 10 μL CCK-8 solution was added to each well and incubated in an incubator for 3 h. Absorbance was measured at 450 nm using a multifunctional microplate reader (PerkinElmer, USA). The viability level of RAW264.7 cells was normalized to the ratio of the control group.

### Determination of bacterial growth dynamics

To evaluate the effect of candidate chemical on *Salmonella* growth in LPM, bacterial growth was monitored using a high-performance microbial growth analyzer (MicroScreen HT, Gering, China). Briefly, bacteria were cultured in LB medium to the logarithmic phase (OD_600_ ~0.25) and then transferred to LPM (1 mL final volume) supplemented with 25, 50, or 100 μg mL^−1^ candidate chemical, whereas the control group received an equivalent volume of PBS. The inocula were incubated at 37°C, with shaking at 180 rpm for 12 h, and OD_600_ was automatically recorded at 30 min interval.

### Time-dependent killing assay

To obtain enough biomass for time-dependent killing assay, the bacteria were first grown in LB to exponential phase, then transferred to LPM (2 mL final volume) at a final concentration of approximately 1 × 10^6^ CFU mL^−1^. Subsequently, the bacteria were exposed to 1/2 MIC of CS with or without the candidate chemical. Then, the matrices were placed in an incubator at 37°C without shaking, and 100 μL aliquots were collected at 0, 3, 6, 9, and 24 h intervals. The collected samples were then enumerated by counting the colony formation units on agar plates.

### Resistance development

The *Salmonella* Typhimurium ATCC 14,028 strain and other two CS-resistant isolates (15E164 & 15E341, Table S2) were selected for resistance development assay, where they were aliquoted into 1 mL LPM (in an Eppendorf tube, 3 biological replicates for each group) medium containing 1/4 MIC of CS with or without candidate chemical. The mixture was incubated at 37°C with continuous shaking at 180 rpm for 24 h. After each cultivation, 50 μL from each culture was inoculated into fresh LPM and serially passaged for 21 d, during which the changes in MIC values of the evolved subpopulations of each strain were regularly monitored using protocol mentioned above in 96-well microtiter plates.

### Scanning electron microscopy (SEM)

The SEM was performed to analyze morphological changes upon treatments using *Salmonella* Typhimurium ATCC 14,028 as proof-of-concept. Briefly, the LB exponential phase bacterial suspension was transferred to LPM and grouped into control group, CS group (1 μg mL^−1^), candidate chemical group and their combination. And then the collected samples were rinsed three times with PBS and then fixed in a 2.5% glutaraldehyde solution at 4°C overnight. Then, the samples were dehydrated using a gradient concentration of ethanol solutions (30%, 50%, 70%, 80%, and 90%) once, and then treated with 100% ethanol twice. The sample was treated by critical point drying (Leica, Germany) after coating by an ion sputtering instrument (Leica, Germany), and finally examined by a field emission scanning electron microscope (Thermo Fisher Scientific, USA).

### Atomic force microscopy (AFM)

The bacteria (*Salmonella* Typhimurium ATCC 14,028) were collected after treatments with CS (1 μg mL^−1^) or in combination with candidate chemical (25 μg mL^−1^). The samples were washed three times with ultrapure water to remove saline before freeze-drying. The freeze-dried bacterial pellets were loaded on a biocompatible substrate for AFM imaging. The aluminum-coated silicon probe AC240TS-R3 (OXFORD, CA) was used, with a symmetrical tip and a spring constant of ~ 2 N m^−1^, to tap the surface of the bacteria in a gas‑phase environment. All AFMs were completed under room temperature, with a scanning rate of 1.5 Hz and a resolution of 256 samples per line. Scanning was performed in AC Air Topography imaging mode. All imaging analyses were performed using Igor Pro (OXFORD, CA). The whole cell scan was fitted to a plane to standardize Z-Height. For the scanning of the bacterial surface topology, the second-order transform was used to flatten the image, and the surface topology was calculated from the cross-sections scanned by these images.

### Bacterial cytoplasmic profiling (BCP)

The *Salmonella* Typhimurium ATCC 14,028 culture was first harvested in rich medium for enough biomass then inoculated into fresh LPM medium cultivation until the exponential phase. Following exposure under CS (1 μg mL^−1^) with or without candidate chemical (25 μg mL^−1^) for 2 h, the bacterial cultures were washed three times with ice-cold PBS buffer and re-suspended. Membrane and nucleus staining were then performed using the dyes FM4-64 (2 μg mL^−1^, T3166, Thermo Fisher Scientific, USA) and DAPI (3 μg mL^−1^, D1306, Thermo Fisher Scientific, USA), with 30 min incubation in the dark. After removing the extracellular probes, the stained bacteria were resuspended and imaged using super-resolution confocal microscopy TCS SP8 STED 3X (Leica Microsystems, Germany).

### Membrane permeability assays

The permeability changes of bacteria’s outer and inner membranes were detected using NPN (HY-W009756, MCE, USA) and PI (HY-D0815, MCE, USA) probes. Briefly, the overnight bacteria (*Salmonella* Typhimurium ATCC 14,028) were transferred into fresh LPM medium (10 μL to 1 mL final volume, 1:100 dilution) and incubated at 37°C with shaking until the exponential growth phase (cultivation time: ~ 4 h). The bacteria were then washed once with PBS and resuspended to an OD_600_ of 0.5 in 5 mM HEPES buffer. After treatment with CS (1 μg mL^−1^) or with candidate chemical (25 μg mL^−1^) for 1 h static incubation, the probe NPN was added at a final concentration of 10 µM. After incubation for 30 min in the dark at room temperature, the fluorescence (λ_ex_ = 340 nm, λ_em_ = 405 nm) was recorded using a multifunctional microplate reader (PerkinElmer, USA) to assess the outer membrane permeability. As to the PI test, overnight culture was transferred into a fresh LPM medium (same protocol) containing CS (1 μg mL^−1^) with or without candidate chemical (25 μg mL^−1^). After washing once with PBS and resuspending (in PBS), a final concentration of 10 nM PI was added and incubated at 37°C in the dark for 15 min static incubation. The fluorescence emission intensity was recorded using a multifunctional microplate reader (PerkinElmer, USA), with an excitation wavelength of 535 nm and an emission wavelength of 615 nm for the detection of PI.

### Cytoplasmic leakage assay

Overnight cultures of *Salmonella* Typhimurium ATCC 14,028 were inoculated in fresh LPM medium (100 μL to 10 mL final volume, 1:100 dilution) containing 1 μg mL^−1^ CS, and incubated with or without candidate chemical (25 μg mL^−1^) for 4 h static incubation at 37°C. Following the treatment, the supernatant of each treatment was obtained by centrifuging at 3400 *g* for 5 min, and the DNA and protein leakages in the supernatants were determined using a spectrophotometer (Thermo Fisher Scientific, USA) according to a previous protocol with minor modifications [24]. Briefly, the leaked DNA and protein contents in supernatants were estimated at 260 and 280 nm respectively. In addition, the leakage of K^+^ was detected with a similar protocol yet in a K^+^-deficient LPM medium using a flame photometer FP6410 (LABO-HUB, China).

### ROS determination by flow cytometry

Overnight cultures of *Salmonella* Typhimurium ATCC 14,028 were washed three times with PBS and resuspended in fresh LPM medium at a density of approximately 5 × 10^7^ CFU mL^−1^. The bacterial suspension was then treated with CS (1 μg mL^−1^) with or without candidate chemical (25 μg mL^−1^), followed by incubation with the DCFH-DA probe (HY-D0940, MCE, USA) for 3 h. Intracellular reactive oxygen species (ROS) in the treated bacterial cells were subsequently detected using a flow cytometer with a 488 nm excitation laser, and the resulting data were analyzed using FlowJo software (Tree Star, Ashland, USA).

### LPS-binding assay

The LPS-binding of CS was assessed using a BODIPY-TR cadaverine (BC, HY-D1594, MCE, USA) displacement assay performed in the presence or absence of candidate chemical. Briefly, the bacterial suspensions (*Salmonella* Typhimurium ATCC 14,028) grown in LB medium were subcultured (1% inoculum) in LPM medium and co-incubated presence or absence of candidate chemical (3.125 μg mL^−1^) until the logarithmic growth phase was reached. Cells were then washed twice with Tris-HCl buffer (20 mM, pH 7.4, HY-D0227, MCE, USA) and resuspended to an OD_600_ of 0.5. Subsequently, BC probe was added to the bacterial suspension at a final concentration of 2 μM, followed by 4 h of incubation in the dark. After removing the unbound probe by washing, the bacterial suspension was aliquoted (100 μL well^−1^) into a black 96-well plate and mixed with an equal volume of CS at varying concentrations. Following 30 min of incubation at 37°C, fluorescence intensity was measured using a multimode microplate reader at excitation/emission wavelengths of 580/620 nm. The displacement efficiency of CS was quantified by calculating the relative fluorescence change (%ΔF) as follows:

%ΔF(AU) = (F_obs_-F_0_)/(F_100_-F_0_) × 100

In the formula, the F_obs_ represents the observed fluorescence at a given CS concentration, F_0_ is the initial fluorescence of BC in the absence of CS (negative control), and F_100_ corresponds to the fluorescence intensity of BC bound to bacterial cells following treatment with 10 μg mL^−1^ polymyxin B (positive control).

### Lipid a profiling

The bacteria (*Salmonella* Typhimurium ATCC 14,028) were cultured in LPM medium containing PBS, CS (1 μg mL^−1^) or candidate chemical (25 μg mL^−1^) for 24 h for sufficient biomass (to gain enough biomass, the bacteria were incubated in 800 mL medium), then the lipid A was extracted following the previous method established by the Melander group [25]. Briefly, the cell pellets were re-suspended in a monophasic Bligh-Dyer buffer (chloroform/methanol/water, 1:2:0.8, v/v/v) and subjected to magnetic stirring at room temperature for 1 h. The mixture was then centrifuged at 2000 × g for 30 min, and the solid precipitate at bottom was collected. The collected precipitate was re-suspended in 27 mL of sodium acetate buffer (12.5 mM, pH 4.5, S818278, Macklin, China), transferred to a clean glass vial, and boiled for 30 min. After cooling to room temperature, 30 mL of chloroform and 30 mL of methanol were added, followed by vigorous mixing. The solution was centrifuged at 2000 × g for 30 min to achieve phase separation. The lower organic phase was carefully collected, transferred to a rotary evaporation flask, and dried under reduced pressure. The dried lipid A was then redissolved in 5 mL of chloroform/methanol (4:1, v/v). For MALDI-TOF MS analysis, 0.5 μL of the sample was immediately spotted onto the MALDI target plate and overlaid with 0.5 μL of 10 mg mL^−1^ α-cyano-4-hydroxycinnamic acid (CHCA, HY-107641, MCE, USA) matrix. Mass spectra were acquired in linear mode using a MALDI-TOF mass spectrometer (SHIMADZU, Japan).

### RNA isolation and RT-qPCR

The bacteria (*Salmonella* Typhimurium ATCC 14,028) were treated by either single CS (0.5 μg mL^−1^), candidate chemical (3.125 μg mL^−1^) or their combinations in LPM. After treatments of 60 min, total RNA was extracted from the treated bacterial cells using the OMEGA Total RNA Kit I (Omega, China) according to the manufacturer’s instructions. The samples were subjected to DNase treatment prior to further test, whose quality was confirmed using NanoDrop (Thermo Fisher Scientific, USA) prior to further assays to ensure no DNA contaminations. Subsequently, reverse transcription was performed using 1 μg of total RNA with the Goldenstar RT cDNA Synthesis Kit (Tsingke Biotechnology, China). Real-time quantitative polymerase chain reaction (RT-qPCR) was conducted using SYBR Master Mix (Vazyme, China). The primer sequences used for RT-qPCR are listed in Table S3. Gene expression levels were calculated using the 2^–ΔΔCt^ method.

### Transcriptional reporter assay

The pUC-LuxCDABE plasmid carrying the promoter of the target gene was transformed into wild-type or mutant *Salmonella* strains (Table S4). Briefly, the pUC19 plasmid was employed as the backbone vector for the construction of a bioluminescent reporter system. The promoter regions of target genes *arnT* and *eptA* were separately fused with the bacterial luciferase operon LuxCDABE, yielding two reporter plasmids designated P_*arnT*_-Lux and P_*eptA*_-Lux, respectively. Bacteria were grown in LB medium to the exponential phase (OD_600_ ~0.25), then subcultured into LPM medium (1:100 transfer, in black walled plates). Then the matrices were incubated with CS (1 μg mL^−1^) in the presence or absence of 25 μg mL^−1^ candidate chemical at 37°C for 12 h, during which luminescence and OD_600_ values were monitored at 30 min interval. The plates were shaken for 5 s before each reading. Luminescence, expressed as relative light units (RLU), was normalized to the corresponding OD_600_ readings.

### Phos-tag assay

Phos-tag assays were performed to determine the phosphorylation status of PmrA as previously described with minor modifications [21]. Briefly, the *pmrA* gene fragment was fused to an HA tag and cloned into the pBAD24 backbone vector to generate the recombinant plasmid pBAD24:*pmrA*-HA. The fidelity of the recombinant construct was validated by Sanger sequencing. The *S*. Typhimurium 14,028 carrying pBAD *pmrA*-HA (Table S4) was grown in an LPM medium containing 0.005% arabinose, in the presence or absence of candidate chemical until the exponential phase. Subsequently, the bacterial cell pellets were harvested by centrifugation at 4°C, subsequently washed twice with precooled Tris-HCl buffer (10 mM, pH 6.8), followed by cell lysis on ice. Each lysate of 120 µL was mixed with 60 µL of 3 × SDS loading buffer and heated shortly on a thermostatic metal bath (Shanghai Yiheng, China). Following heating, equal volumes of the samples (10 µL) were loaded onto an SDS-PAGE gel supplemented with Phos-tag^TM^ acrylamide (50 μM, AAL-107, Wako) and MnCl_2_ (100 μM). Electrophoresis was conducted at 4°C to separate phosphorylated and non-phosphorylated forms of PmrA-HA, which were later transferred onto polyvinylidene difluoride (PVDF) membranes. After membrane blocked by 5% skim milk, PmrA-HA was probed with Mouse anti HA-Tag mAb (AE008, Abclonal, China). Protein bands were visualized using enhanced chemiluminescence (P10100, NCM, China), and phosphorylation level was calculated as the intensity of the phosphorylated PmrA band relative to the total PmrA signal.

### Measurements of intracellular magnesium, pH, and iron

Mag-Fura-2 AM (MKBio, China) dye was used to measure intracellular Mg^2 +^ in bacteria. The bacterial cultures were incubated with or without candidate chemical (25 μg mL^−1^) treatment at 37°C for 6 h before washing and resuspending in LPM. After washing with PBS, the cells were loaded with 5 μM dye and incubated for 1 h at room temperature in the dark. Subsequently, the cells were washed three times and further incubated for 30 min to ensure complete de-esterification of the intracellular dye. Fluorescence of Mag-Fura-2 was then measured using a multimode microplate reader at excitation/emission wavelengths of 380/509 nm.

The intracellular pH was examined with BCECF AM (S10006, Beyotime, China) dye. Bacteria was incubated with or without candidate chemical (25 μg mL^−1^) treatment at 37°C for 6 h after washing and re-suspending in LPM and then incubated with 5 μM dye for 30 min. The fluorescence intensity was also recorded by a microplate microtiter plate reader at excitation/emission wavelengths of 488/535 nm.

The intracellular iron profiling of bacteria was determined using the Iron Content Assay Kits (BC5315, Solarbio, China) with modifications. In brief, after washing and resuspending the cells with LPM medium, the cells were incubated with or without candidate chemical (25 μg mL^−1^) for 6 h (37°C), then washed three times with PBS and sonicated, and then the iron contents were determined using the kit.

### The determination of iron forms

Following co-incubation with the candidate chemical (25 μg mL^−1^), bacterial cell pellets were harvested and re-suspended in ddH_2_O, then subjected to lysis by ultrasonication. The cell lysates were then subjected to iron quantification using a modified protocol of a previously described method [21]. Total iron content was measured using a Total Iron Colorimetric Assay Kit (E-BC-K772-M, Elabscience, USA), while ferrous iron (Fe^2 +^) levels were determined with a Cellular Ferrous Iron Colorimetric Assay Kit (E-BC-K881-M, Elabscience, USA).

### Iron reduction assay

To assess the iron-manipulating capacity of the selected compounds, a modified Ferrozine-based assay was performed for determining whether candidate chemical could directly alter iron speciation through its chemical reducing activity. Ferric chloride (FeCl_3_) was dissolved in LPM medium to a 100 μM concentration. After incubating with candidate chemical (25 μg mL^−1^) for 10 min, the Ferrozine reagent was added, with hydroxylamine hydrochloride (NH_3_OHCl, 100 μΜ) as the positive control. Absorbance was measured at 562 nm using a multifunctional microplate reader (PerkinElmer, USA).

### Isothermal titration calorimetry

To elucidate the molecular interaction mechanism between iron ions and selected compounds, a comprehensive thermodynamic investigation was conducted using isothermal titration calorimetry (ITC, TA Instruments, USA). All measurements were performed at 25°C with high-precision microcalorimetry. During the titration, the Fe^3+^ solution was automatically injected into the sample cell for 25 consecutive aliquots, with 180-s intervals, to ensure complete thermal equilibration. The raw thermogram data were processed by NanoAnalyze software (TA Instruments), employing a single-site binding model to determine the dissociation constant (Kd).

### Measurement of lipid peroxidation in bacteria

The lipid peroxidation in bacteria was determined based on a previously published protocol with modifications [26]. Briefly, bacteria were challenged by CS (1 μg mL^−1^) with or without candidate chemical (25 μg mL^−1^) in LPM. After treatment, the bacterial cells were washed twice with ice-cold Tris-HCl buffer (50 mM, pH 8.0) containing 20% (w/v) sucrose. Subsequently, 0.2 mL of lysozyme solution (5 mg mL^−1^ in 0.25 M Tris-HCl, pH 8.0) and 0.4 mL of EDTA (0.25 M, pH 8.0) were added to the cell suspension, followed by incubation at 37°C with shaking (200 rpm) for 30 min. After incubation, the cell pellet was re-suspended in citrate buffer (sodium citrate dihydrate: 25.703 g, citric acid: 2.421 g; in 1 L water, pH = 7) and co-incubated with C11-BODIPY 581/591 at 37°C with shaking for 30 min. Flow cytometry was performed using the BD 488 nm channel, and data acquisition was stopped after collecting approximately 10,000 events. The obtained data were analyzed using FlowJo software (Tree Star, Ashland, USA).

### Determinations of ferroptotic byproducts and GSH depletion

The 4-hydroxynonenal (4-HNE) and malondialdehyde (MDA) were detected as the typical ferroptotic byproducts in this study. Before determination, the bacteria were first incubated in LPM medium (1 mL final volume) and cultivated until CS challenge (OD_600_ ~0.25). Following challenging by CS (1 μg mL^−1^) with or without candidate chemical (25 μg mL^−1^) at 37°C for 6 h. Then, the challenged bacteria were collected by centrifugation at 12,000 g and resuspended in PBS. The yielded bacterial cell pellets were subjected to sonication to collect the bacterial lysate, which was used for the determination of the intracellular 4-HNE in bacteria using LC-MS/MS according to a prior method with minor modifications [27]. With the same bacterial lysate prepared as mentioned above, another lipid peroxidation byproducts of the bacteria, MDA, was measured using MDA commercial assay kit (A003-1–2, Nanjing Jiancheng, China). This kit allows measurement of MDA using thiobarbituric acid (TBA), which reacts with MDA to form pink colored complex can be detected under absorbance at 540 nm. The glutathione was assayed with GSH and GSSG Assay Kits (S0053, Beyotime, China).

### Genomic DNA degradation

Bacterial cultures were treated with CS (1 μg mL^−1^) alone or in combination with candidate chemical (25 μg mL^−1^) and then collected for analysis. After treatments, the bacterial cells were broken by lysis buffer and heating to ensure consistent sample handling, then the bacterial genomic DNA was extracted using the Bacteria Genomic DNA Extraction Kit (TaKaRa MiniBEST, TaKaRa BIO INC, Beijing). The genome concentration was determined using the Nanodrop 2000 spectrophotometer (Thermo Fisher Scientific, USA) and normalized. A preliminary evaluation was performed by loading 700 ng of gDNA into a 0.8% agarose gel for gel electrophoresis. ImageJ was used for quantifying DNA degradation.

### Animal trial and in vivo safety evaluation

Female C57BL/6 mice aged 6–8 weeks were orally infected with *Salmonella* at a dose of 10^6^ CFU mL^−1^ over 4 consecutive days while receiving drug treatment, which was optimized on the basis of our previous publication [21]. The infected mice were randomly divided into four groups: each group consisting of 10 mice (*n* = 10), including a control group, a candidate chemical (10 mg kg^−1^) group, a CS (5 mg kg^−1^) group, and a combined group using both candidate chemical and CS (10 mg kg^−1^ candidate chemical +5 mg kg^−1^ CS). The drug cocktail was intraperitoneally injected at the appropriate dose for 5 d [21,28,29]. Before sampling, the mice were euthanized after anesthesia with isoflurane. Bacterial load counts were performed on liver, spleen, kidney, colon, and fecal samples, and the survival status of mice in each group was continuously monitored throughout the experiment. The same administration process was applied to uninfected mice, and their organs were collected post-treatment. Tissue sections were then examined for changes using HE staining.

### Statistical analysis

GraphPad Prism 10.4 software was used for statistical analysis, and the results were expressed as mean ± Standard Deviation (mean ± SD). Unless otherwise stated, an unpaired *t*-test or Mann-Whitney U test (*p < 0.05, **p < 0.01, ***p < 0.001, ****p < 0.0001) was used to evaluate the statistical significance of the comparison.

## Results

### BAI potentiates CS activity against Gram-negative pathogens in macrophage-mimicking conditions

**Figure 1.**
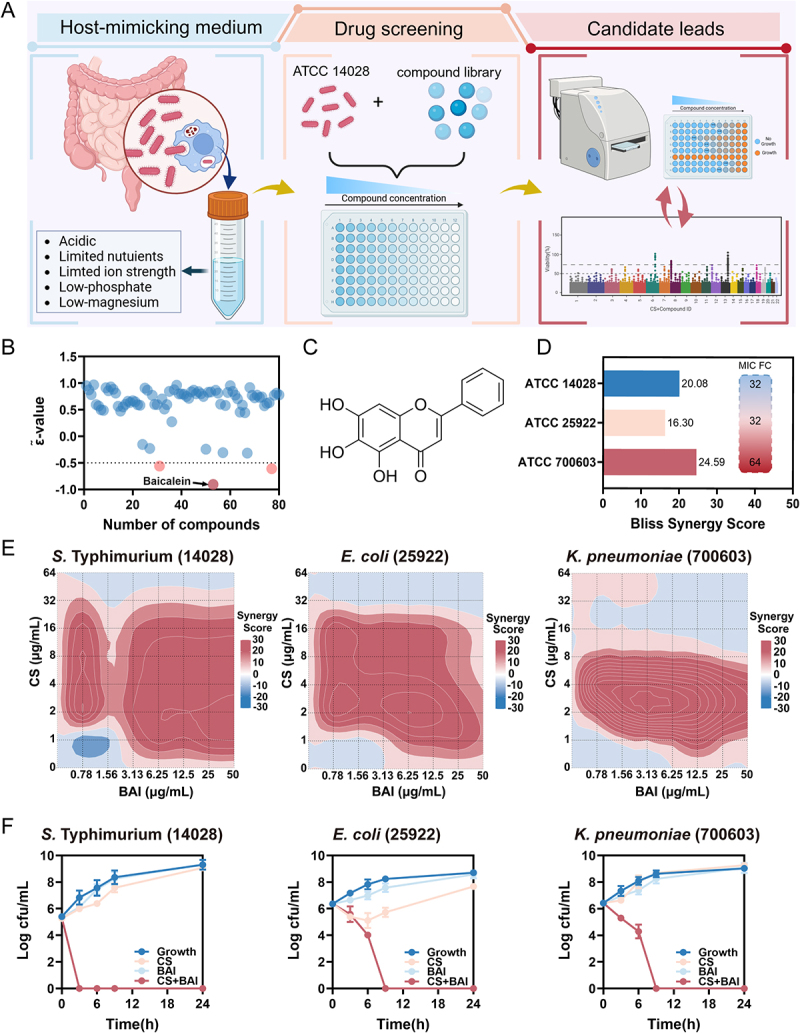
BAI potentiates CS potency under host-mimicking conditions. (A) Schematic illustration for chemical screening based on a low-phosphate, low-magnesium medium (LPM); (B) High throughput screening of CS potentiators (the $\tilde{\varepsilon }$ value, calculated based on the relative bacterial growth under monotherapy or combination treatment, was used to characterize the interaction between CS and the screened compounds, where BAI was identified as the top candidate to enhance CS efficacy); (C) Chemical structure of BAI; (D) Bliss synergy scores between CS and BAI against a panel of Gram-negative pathogens (*S*. Typhimurium: ATCC 14,028, *E. coli*: ATCC 25,922, *K. pneumoniae*: ATCC 700,603, the Bliss score calculated by the Synergyfinder 3.0); (E) The synergism landscape of BAI and CS against Gram-negative pathogens (the combination doses in the red area indicate a synergistic response, while those in blue indicate indifference, as calculated by Synergyfinder 3.0); (F) The time-killing assays show that BAI enhances the bactericidal activity of CS against Gram-negative pathogens (bacteria from the exponential phase were challenged with 2 μg mL^−1^ CS, with or without 25 μg mL^−1^ BAI) Data in (D) and (F) represented three biological replicates and are shown as mean ± SD. Schematics in panels a were created with BioRender.com (https://biorender.com).

The microenvironments of infection sites have been reportedly found to be different from the standard rich media that is widely applied for *in vitro* AST (Antimicrobial Susceptibility Testing) [30]. These microenvironments are known to present unique traits in pH, ion strength, and nutrient availability, which are together reciprocally coupled with altered antibiotic susceptibility for the bacteria therein [31–33]. A well-documented example is that infection microenvironments stimulate the adaptive resistance to deprive the CS sensitivity in Gram-negative bacteria as a general response paralleled by the nutritional limitation, although species-specific differences in regulatory pathways may exist [34–37]. In this regard, we initiated the primary screening for CS potentiators in LPM, a macrophage-relevant medium whose acidic pH and low Mg^2+^ availability partially recapitulate key features of the intramacrophage environment [23,38,39] (Figure 1(A)). Under such host-relevant condition, the indicator strain (*Salmonella* Typhimurium strain ATCC 14,028) as well as a panel of common Gram-negative bacteria were observed more resistant to CS comparing that of it in rich CAMH medium (Table S2). In this protocol, potentiation was indicated by the $\tilde{\varepsilon }$ value, which was calculated based on normalized bacterial growth following exposure to a single drug or a drug combination (see the Methods section for the detailed computational procedure). Through an in-house screening based on a chemical library on lab stock, 3 hits (3/80, 3.75%) were identified to promote growth inhibition of CS, wherein a phytochemical, baicalein (BAI) was found prominently potent (Figure 1(B) & Table S1). This molecule is originally isolated in *Scutellaria baicalensis* and presents a classic flavone structure (5,6,7-trihydroxyflavone, Figure 1(C)), holds desirable biosafety (Fig. S1) and actively modulating many biological processes, such as oxidative response, tumor immunity, and cell proliferation in eukaryotes [40]. However, this natural molecule generally exerts negligible impact on viability of Gram-negative pathogens like *Salmonella* (Fig. S2), however its potential as an antibiotic potentiator has not been reported. To affirm the synergistic interaction between CS and BAI, three CS-susceptible standard Gram-negative bacterial strains (*Salmonella* Typhimurium ATCC 14,028, *Escherichia coli* ATCC 25,922, and *Klebsiella pneumoniae* ATCC 700,603) were then subjected to multi-dose drug combination assays and analyzed using Synergyfinder [41]. As shown in Figure 1(D), the average synergy scores (as indicated by the Bliss Independence Model, Bliss score) were observed ranging from 16.30 to 24.59, indicating a promising synergistic action between BAI and CS. The 2D counter plots based on the dose-response of BAI-CS combination demonstrated a desirable synergy landscape pronounced at a wide spectrum of concentrations on selected strains (Figure 1(E)). These encouraging results were reconfirmed by the classic checkerboard assay, where the fractional inhibitory concentration index (FICI) values ranging from 0.0312 ± 0 to 0.25 ± 0.0156 and MIC decreased up to 64-fold (Fig. S3-4 & Table S2). In view of reinforcing the understanding toward the adjuvant property of BAI, we next performed a time-kill dynamic on the aforementioned strains. The growth of all tested strains was barely inhibited by monotherapy of either BAI or CS, suggesting less optimal efficacy of CS under host conditions. In contrast, the combination with BAI maximized the CS activity to significantly reduce bacterial load, and completely sterilize the bacteria within 9 h (Figure 1(F)). Overall, these results indicate that BAI is a promising adjuvant to CS under host-mimicking conditions, which might serve as an important player in tackling the infections caused by Gram-negative pathogens.

### BAI restores CS efficacy against resistant isolates and reduces emergence of resistance

**Figure 2.**
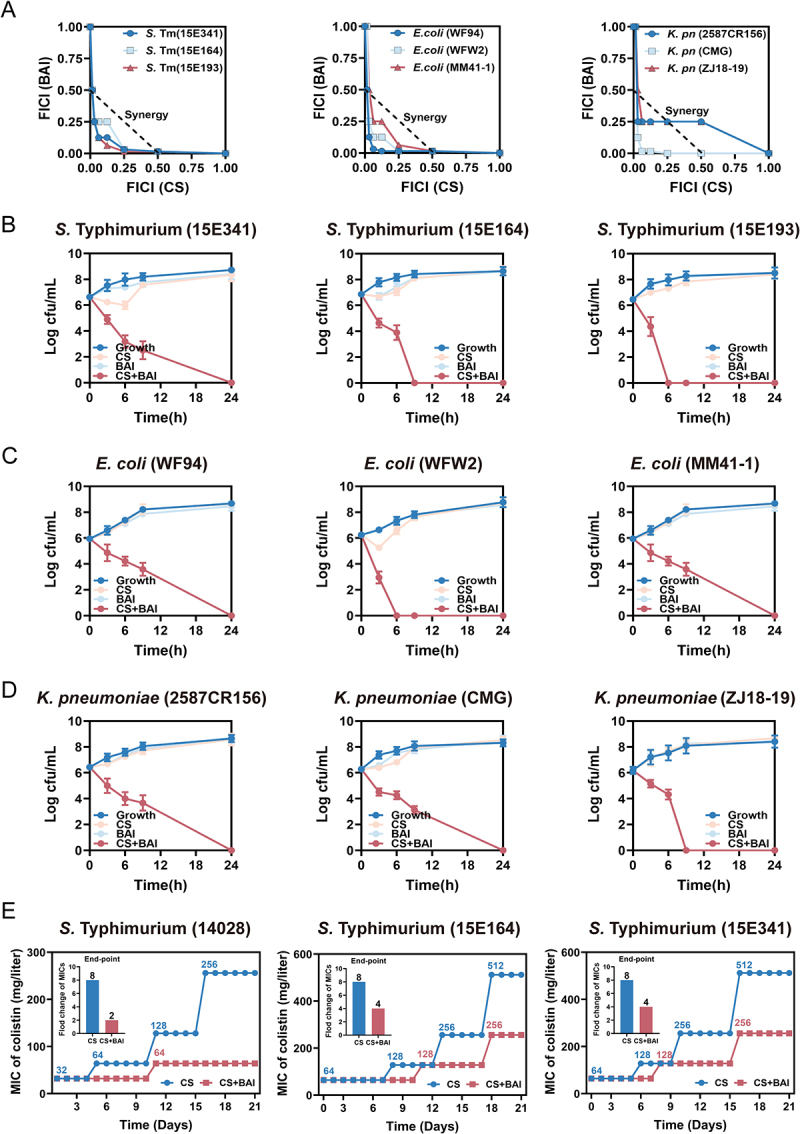
BAI restores CS activity against resistant isolates under LPM. (A) Isobolograms for the combination of BAI and CS against CS-resistant isolates harbored *mcr* allele or *mgrB* mutations (*S*. Typhimurium: 15E341, 15E164, 15E193; *E. coli*: WF94, WFW2, MM41-1; *K. pneumoniae*: CMG, ZJ18-19, 2587CR156); BAI potentiates CS killing on the CS-resistant isolates of *S*. Typhimurium (B, strain 15E341, 15E164, 15E193), *E. coli* (C, strain WF94, WFW2, MM41-1), and *K. pneumoniae* (D, strain CMG, ZJ18-19, 2587CR156), where the CS (4 μg mL^−1^) was applied with or without BAI (25 μg mL^−1^); (E) Evolution of CS resistance in both CS-sensitive (*S*. Typhimurium ATCC 14,028) and -resistant bacteria (*S*. Typhimurium 15E341 and 15E164) treated with or without BAI (CS: 8 μg mL^−1^, BAI: 25 μg mL^−1^, serially passaged in Eppendorf tubes with three biological replicates; the MIC increment at each breakpoint is indicated in the line plot, and the fold-change in MIC at the end of the experiment is presented in the bar plot within the figure). Data in (A-E) represented three biological replicates and are shown as mean ± SD.

Besides the model strains, it is also important to study whether BAI is able to enhance CS activity against the CS-resistant strains for fully establishing its potential as a viable CS adjuvant. Thus, we determined the synergistic interaction between BAI and CS on nine selected isolates from three major Gram-negative species that are resistant to CS by either chromosomal or plasmid-conferred mechanisms. The nine selected strains included *mcr*-1-expressing *S*. Typhimurium strains (15E341, 15E164, and 15E193), *E. coli* (WF94, WFW2, and MM41-1), *K. pneumoniae* (CMG), as well as *K. pneumoniae* strains ZJ18-19 and 2587CR156, both with *mgrB* inactivation. As depicted in the isobolograms (Figure 2(A)), there was a notable synergistic effect between BAI and CS, with fractional inhibitory concentration indices (FICIs) ranging from 0.016 ± 0 to 0.292 ± 0.191 (Fig. S5) and reduced MIC up to 64-fold for all tested strains (Fig. S6 & Fig. S7 & Table S2). To further evaluate the combination of BAI and CS against the aforementioned resistant strains, we conducted time-killing experiments. Notably, the results showed that neither BAI nor CS alone completely eradicated the candidate CS-resistant pathogens, whereas the incorporation of BAI significantly accelerates the pathogen clearance by CS, thereby completely eliminating the bacteria in 6–24 h (Figure 2(B-D)). The previous reports highlighted that exposure to CS at a bactericidal amount generally triggered the selection of resistance [11]. To this point, we wondered whether the combination with BAI is capable of interfering with these resistance selections. Hence, both CS-sensitive and CS-resistant strains (*S*. Tm ATCC 14,028, 15E164, and 15E341) were used as representatives for the serial passage in the presence of CS (1/4 MIC) with or without BAI. The results demonstrated that the incorporation of BAI minimized the resistance emergence to CS throughout the experiments, whereas CS alone rapidly led to an increase in MIC, with strain 14,028 and strains 15E164 and 15E341 exhibiting up to an 8-fold increase (Figure 2(E)). Taken together, these results jointly indicate that BAI can be used as an effective adjuvant to potentiate CS activity against CS-resistant strains and prevent the selection of CS resistance in the macrophage-mimicking conditions.

### BAI promotes membrane actions of CS to destabilize envelope integrity

**Figure 3.**
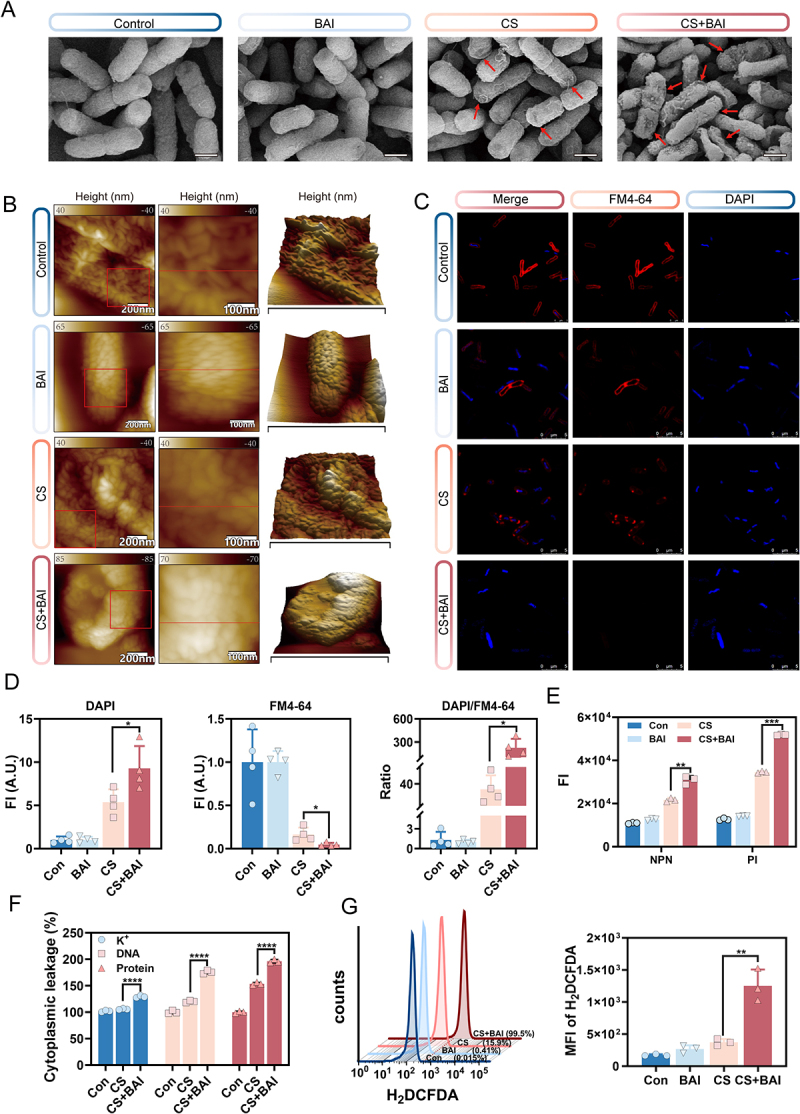
BAI promotes the membrane action of CS to induce toroidal pores and reactive species. (A). Morphological changes of *Salmonella* under treatment of CS (1 μg mL^−1^), BAI (25 μg mL^−1^) and their combinations as shown by SEM analysis (scale bar: 500 nm); (B). Topological changes of bacteria treated by CS (1 μg mL^−1^) and their combinations as indicated by the AFM analysis. The scale bar in the left column of the AFM image represents 200 nm, and the bar scale in the middle is 100 nm; (C). Bacterial cytoplasmic profiling on bacteria treated by CS (1 μg mL^−1^) with or without BAI (red: FM4-64; blue: DAPI; scale bar: 5 μm, 25 μg mL^−1^); (D). Quantitative analysis of bacterial cytological profiling unveiled increased toroidal pore formation in bacterial cells treated by CS + BAI; (E). Permeability change of bacteria treated by CS (1 μg mL^−1^) with or without 25 μg mL^−1^ BAI (FI: fluorescence intensity; the permeability of outer membrane was indicated by probe 1-N-phenylnaphthylamine, and the permeability of inner membrane was indicated by the probe propidium iodide); (F). Cytoplasmic contents leakage assessments of bacteria treated by CS (1 μg mL^−1^) in combination with or without BAI (25 μg mL^−1^); (G). The measurements of reactive oxygen species generation caused by CS (1 μg mL^−1^), BAI (25 μg mL^−1^) and their combination (the total reactive species generation was assayed by the H_2_-DCFDA probe). Data in (D-G) represented three biological replicates; all data were shown as mean ± SD. The unpaired *t*-test was used for the statistical analysis where * = *p* < 0.05, ** = *p* < 0.01, *** = *p* < 0.001, **** = *p* < 0.0001.

Given the therapeutic potential of pairing BAI with CS, we next sought to explore the mechanism for their synergy. Since BAI exhibited the strongest potentiating activity on CS against *Salmonella* species, the strain ATCC 14,028 was used as the proof-of-concept for following mechanistic investigation. Although the mechanisms for CS to kill bacteria are not fully explored, its membrane actions to directly target the negatively charged lipid A of lipopolysaccharide (LPS) underpin the primary mechanism of CS. In this view, we first probed the morphological alterations of bacteria exposed to CS in combination with or without BAI by scanning electron microscope (SEM) analysis using *S*. Typhimurium ATCC 14,028 as the model. Compared with monotherapy using CS, the combination produced more significant damage to membranes of treated bacteria, with severe superficial blisters and irregular surfaces (Figure 3(A)). To gain in-depth insights into the membrane actions of CS in the presence of BAI, the atomic force microscopy (AFM) was applied to characterize bacterial surface topology. AFM height-profile analysis showed that BAI alone induced a modest change in membrane surface topology, whereas the combination of CS and BAI caused strong undulations in the average surface height in the tested bacteria and generated crevices in the surfaces (Figure 3(B) & S8). These topological changes, together with SEM analyses, underlined the promoted CS action to perturb the bacterial membranes by BAI, which has implications for destroying the membrane integrity and functions as previously documented [42]. To validate the disrupted membrane integrity caused by BAI-CS combination, the bacterial cytoplasmic profiling (BCP) was performed by staining the membranes and nucleoids using FM4-64 and DAPI, respectively [43]. As observed in Figure 3(C,D) the combination destabilized the fluorescence intensity of FM4-64 (red) and enhanced the fluorescence intensity of DAPI (blue), indicating that the CS rapidly generated breaches in the bacterial membranes, which facilitated diffusion of dyes through the membrane. Those breaches, defined as the toroidal pores, are thought to ultimately break the barrier functions of the membrane between cytoplasm and the outer environment [44]. To clarify this, two dyes for the measurement of membrane permeability, NPN and PI, were used to elucidate the alteration in outer and inner membrane barrier functions in response to the addition of the BAI-CS combination. As illustrated in Figure 3(E), significant increases in the fluorescent intensities of NPN and PI were observed following BAI and CS treatment, thus confirming that the addition of BAI enhanced the CS-mediated membrane permeability disruption. As the biological consequence of permeability change, we further observed the leakage of bacterial cytoplasmic contents (relative leakage), including DNA, proteins, and ions (K^+^ as a proof-of-principle) in the bacteria treated by different treatments. Notably, although CS was used at a sublethal concentration, the combination treatment still produced an approximately twofold increase in leakage compared with the control, indicating a marked enhancement of membrane disruption (Figure 3(F)). These changes by BAI-CS combination directly led to drastic generation of reactive species in the tested cells (Figure 3(G)). These results indicate that the CS-BAI combination is associated with enhanced membrane perturbation and increased reactive species accumulation, may together contributing to fast killing of CS in presence of BAI. Collectively, the data above revealed the primary mechanism for BAI in synergizing with CS by promoting its membrane action to induce a cascade of lethal damage, including toroidal pores and reactive species.

### BAI enhances the electrostatic interaction between CS and the membrane by paralyzing PmrAB-dependent LPS profile remodeling

**Figure 4.**
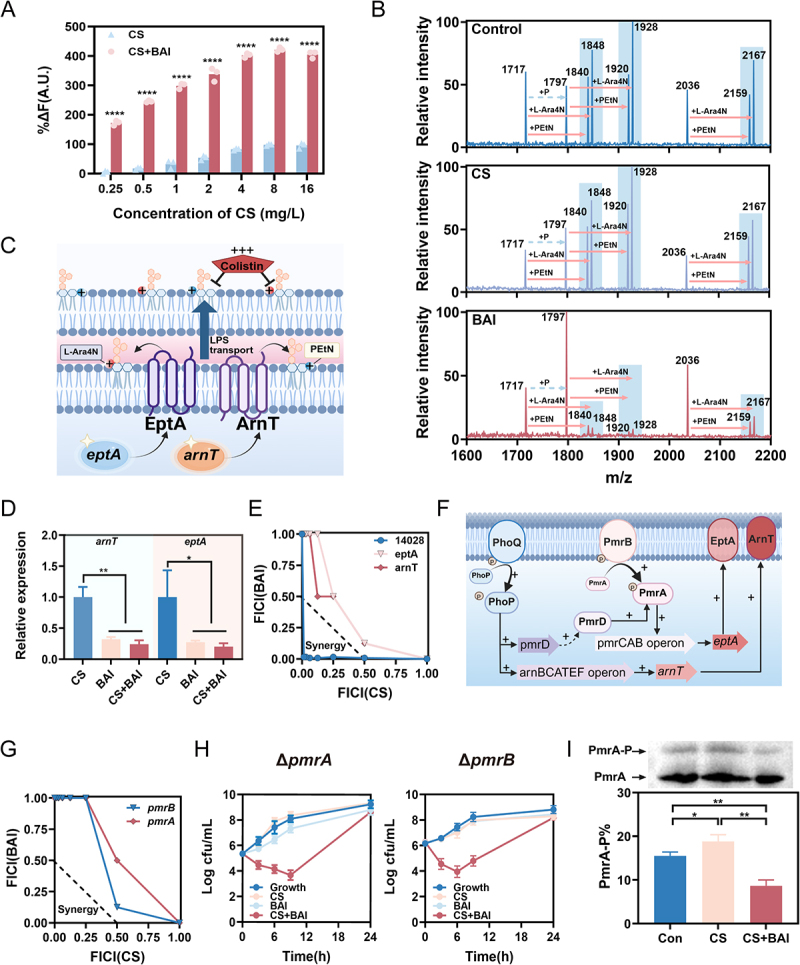
BAI remodels LPS modifications via blockade of PmrA/B signaling to enhance membrane binding and actions of CS. (A) BODIPY-Cadeverine binding assay indicates BAI enhances the electrostatic interaction between CS and bacterial membrane (the bacterial cells were pre-incubated with or without BAI: 3.125 μg mL^−1^) for 12 h, transferred to Tris-HCl buffer supplemented with BODIPY-Cadeverine, and then adding different concentrations of CS, whose fluorescence increases upon enhanced CS to LPS binding and displacement); (B) LPS profiling using MALDI-TOF suggests BAI decreases the cationic modifications on lipid a moieties (the bottom spectrum indicates the lipid a fractions in bacteria were grown in the presence of either CS: 1 μg mL^−1^ or BAI: 25 μg mL^−1^); (C) Schemes for the regulations of LPS modifications in Gram-negative bacteria; (D) Expression of genes (*eptA*/*arnT*) responsible for LPS modifications in the presence or absence of BAI (bacteria were pre-treated by CS:1 μg mL^−1^, BAI: 25 μg mL^−1^ and their combinations in LPM before RNA isolation); (E) Loss of genes for LPS modifications abolished the potentiation of CS by BAI (isobolograms for the combination of BAI and CS against the mutants lacking *eptA* and *arnT*); (F) The bacterial two-component systems are the central hub to govern the LPS modifications; (G-H) Loss of functions in PmrA/B signaling diminishes the synergism between BAI and CS (isobolograms for the combination of BAI and CS against the mutants lacking *pmrA* and *pmrB*, the bacteria were challenged by 1/4 ΜΙC CS (0.5 μg mL^−1^) with or without BAI); (I) BAI blockades the PmrA/B signaling by inhibiting its activation via phosphorylation (phos-tag assay indicates the phosphorylation of PmrA in the presence or absence of BAI, CS:1 μg mL^−1^; BAI: 25 μg mL^−1^) Data in A, D, E and H-I represented three biological replicates and were shown as mean ± SD. The unpaired *t*-test was used for the statistical analysis where * = *p* < 0.05, ** = *p* < 0.01, *** = *p* < 0.001, **** = *p* < 0.0001, ns not significant. Schematics in panels C and F were created with BioRender.com (https://biorender.com).

With confirmed evidence in the primary action of BAI in enhancing membrane actions of CS, we sought to clarify the exact mechanism for the regulation of BAI. Intriguingly, we found that, compared to using CS alone, the BAI-CS combination increased the bacterial surface roughness (Fig. S8). This phenotype has often been observed when antimicrobial peptides are incorporated into bacterial membranes and causes a “crumping” effect owing to expansion of surface area [45]. Thus, it left us a plausible assumption that the BAI might be able to facilitate the binding and incorporation of CS into bacterial membranes. To further confirm this hypothesis, we then assessed the direct LPS-binding ability of CS to bacterial membranes, with or without BAI, with BODIPY TR cadaverine (BC) displacement assay using polymyxin B as a well-established control [46]. The BC probe is quenched when interacting with LPS, and the introduction of CS subsequently displaces BC, increasing its fluorescent signals. As shown in Figure 4(A), in the presence of BAI, BC-mediated fluorescence showed a strong enhancement in a dose-dependent manner, whereas this enhancement was not caused by addition of BAI per se (Fig. S9). This suggested that BAI facilitated CS binding actions to bacterial surfaces, which explained the rationale for enhanced membrane actions of CS in the presence of BAI. It has been reported that the surface charge of bacterial membranes is often correlated with the modifications on LPS in Gram-negative bacteria [47]. To determine whether BAI modulates bacterial LPS profiles, we performed modified MALDI-TOF/MS on untreated bacteria and bacteria treated with either CS or BAI, following a previously established protocol [48]. The data elaborated that BAI treatment resulted in sharp decreases in the peaks for the lipid A with cationic PEtN (phosphoethanolamine) and L-Ara4N (4-amino-L-arabinose) modifications (Figure 4(B)), which were reported to collectively counteract the CS binding in previous publications [49]. In this regard, the remodeled LPS profiles by BAI were conceived to be the biochemical basis for its potentiation of CS activity. In Gram-negative species, these chemical modifications are known to be executed by the EptA and ArnT encoded by the *eptA* and *arnT* genes to offset the unfavorable conditions (Figure 4(C)). Therefore, we reasoned that BAI putatively modulates the expression of these genes to lower the bacterial membrane charge by reducing cationic modifications on LPS. To this end, both RT-qPCR and the bioluminescence reporter assays were performed to monitor the transcription dynamics of these genes. The results indicated that the expressions of both *eptA* and *arnT* were significantly downregulated after the addition of BAI (Figure 4(D) & Fig. S10). To test whether BAI-mediated LPS profiling remodeling was responsible for its synergy with CS, we next examined the interaction of the BAI-CS pairs on the mutants defective in *eptA* and *arnT*. As expected, the loss of *eptA* and *arnT* readily abolished the synergy between BAI and CS (Figure 4(E)). This finding inspired us to further explore the driver that responded to BAI and exerted the regulatory actions on the expression of *eptA* and *arnT*. The prior investigations have documented that these genes are under the stringent governance of bacterial two-component system (TCS) PhoP/Q and PmrA/B (Figure 4(F)). Thus, we hypothesized that such TCS might be a determinant for synergism between BAI and CS by modulating the expression of *eptA* and *arnT*. By determining the activity of the BAI-CS combination in mutants with defects in *phoP*, *phoQ*, *pmrA*, and *pmrB*, it was found that the deletion of PhoP/Q TCS only dampened (Fig. S11), but the absence of PmrA/B TCS fully abolished the synergistic interaction between BAI and CS (Figure 4(G,H)). This suggested inactivation of PmrA/B by BAI defined its activity for LPS remodeling and CS potentiation. Considering TCSs are the systems that are only activated to regulate gene expressions when the response regulator (PmrA in this case) is phosphorylated by its cognate histidine kinase, we next employed the Phos-tag assay to investigate the impact of BAI on PmrA phosphorylation under CS exposure, thereby enabling evaluation of whether BAI modulates PhoPQ signaling during CS challenge. The activation of PmrA/B was modestly primed by single CS treatment yet significantly blocked by BAI, as the PmrA protein was restricted in its unphosphorylated form after combination (Figure 4(I)). In concert with this, the addition of BAI further decreased transcription of either *pmrA* or *pmrB*, as a result of inactivation of the PmrA/B system (Fig. S12). These findings supported that BAI was specifically involved in the cascade regulation of PmrAB-mediated LPS remodeling to synergize with CS. Altogether, these results suggested that BAI inhibited the PmrA/B-dependent LPS profile remodeling to promote electrostatic interaction between CS and bacterial surface, which functioned as a central hub for BAI-mediated CS potentiation.

### Orchestration of cellular labile iron defines BAI-mediated inactivation of the PmrA/B system

**Figure 5.**
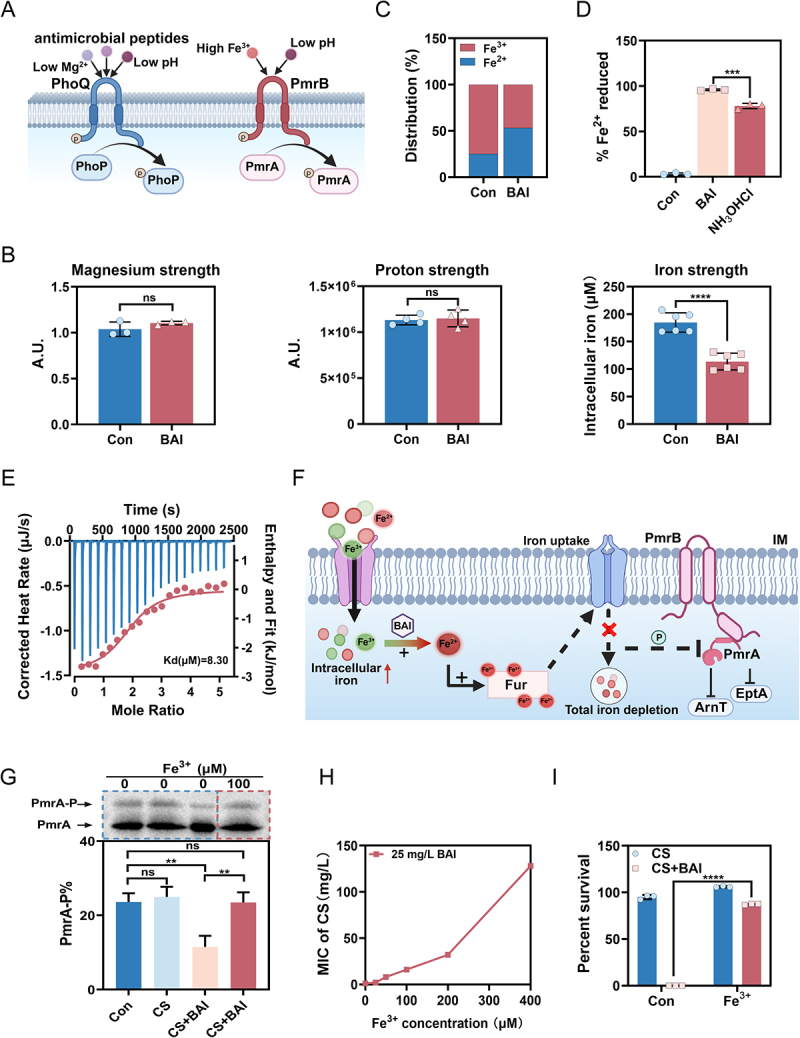
BAI inactivates the PmrA/B system by manipulating the bacterial cellular labile iron. (A) Biological stimuli that modulate two-component systems for LPS modifications; (B) Total magnesium, proton and iron strength measurements in bacteria treated with or without BAI (25 μg mL^−1^); (C) The compositionally changes of bacterial labile iron pool in response to BAI (25 μg mL^−1^) treatment; (D) Iron reduction from ferric to ferrous form in bacteria treated by BAI (25 μg mL^−1^); (E) ITC analysis demonstrates that BAI directly binds to iron; (F) Proposed mode of action of BAI to orchestrate the cellular labile iron; (G) Phosphorylation of bacterial PmrA/B system in the presence of exogenous ferric iron (blue box: without ferric iron supplementation; red box: with ferric iron supplementation; CS:1 μg mL^−1^, BAI: 25 μg mL^−1^); (H-I) Exogenous addition of ferric iron abrogates the enhanced CS activity by BAI. Data in (C-D) and (G-I) represented three biological replicates and were shown as mean ± SD. The unpaired *t*-test was used for the statistical analysis where * = *p* < 0.05, ** = *p* < 0.01, *** = *p* < 0.001, **** = *p* < 0.0001, ns not significant. Schematics in panels A and F were created with BioRender.com (https://biorender.com).

The results above suggested that shutting off PmrA/B-dependent LPS remodeling defined the synergy between BAI and CS; it was of great interest to decipher the underlying mode of action of how BAI blocks PmrA/B activation. In a general context of TCS signaling, the cognate histidine kinase initiates the signal transduction by phosphorylating the regulator protein after sensing specific cues. In this case, the sensor kinase PmrB is known to respond to the availability of several ions, including protons, magnesium, and iron (Figure 5(A)) [50]. The cytoplasmic proton and magnesium were found stable using the specific probes, yet the total intracellular iron was significantly reduced after BAI treatment (Figure 5(B)). In tandem with the sharp reduction in the total iron amount, the addition of BAI turned the ferrous iron into the dominant form instead of ferric iron (Figure 5(C)). These changes implied that BAI shifted the cellular labile iron pool within bacteria since the iron generally occurs in stable ferric form in most biologically relevant conditions [51]. With the precedent that BAI is a potent iron modulator under physiological conditions in mind [52], we proposed a hypothesis where the BAI disrupts the cellular iron biology by manipulating the labile iron pool to block the PmrB sensing and PmrA activation, thereafter inactivating the PmrA/B-dependent LPS modifications. Therefore, in this case, BAI was assumed to modulate the intracellular iron by form transition rather than chelation, although the previous investigations mainly focused on its chelating property [52]. This assumption was validated by the *in vitro* iron form analysis, which showed that the ferric iron was massively reduced to ferrous form by BAI in a dose-dependent manner (Figure 5(D)). In addition, the isothermal titration calorimetry (ITC) analysis was performed to determine the interaction between the ferric iron and BAI, in which BAI had a high affinity for ferric ion to convert them into ferrous form (Figure 5(E)). This might explain the rationale behind the drop in total iron content as the ferrous iron in excess interacts with the Fur (ferric uptake regulator) as a cofactor, thereby limiting the iron uptake to drain the iron pool in BAI-treated bacteria (Figure 5(F)). Given the importance of ferric iron in PmrA/B activation and subsequent LPS modification, such iron-based regulation was conceivably to modulate bacterial susceptibility to CS. This was solidly supported by the data that the exogenous ferric iron restored the phosphorylation of the PmrA regulator protein (Figure 5(G)). And as an expected biological consequence, the addition of ferric iron not only diminished the synergism between BAI and CS but also maximally rescued the bacterial survival under the BAI-CS combination (Figure 5(H,I)). Jointly, these results showed that BAI dysregulated the cellular labile iron within bacteria to lessen the accessible ferric iron, which was necessary for PmrA/B activation.

### Cellular iron dyshomeostasis augments CS killing by inducing ferroptotic-like damage

**Figure 6.**
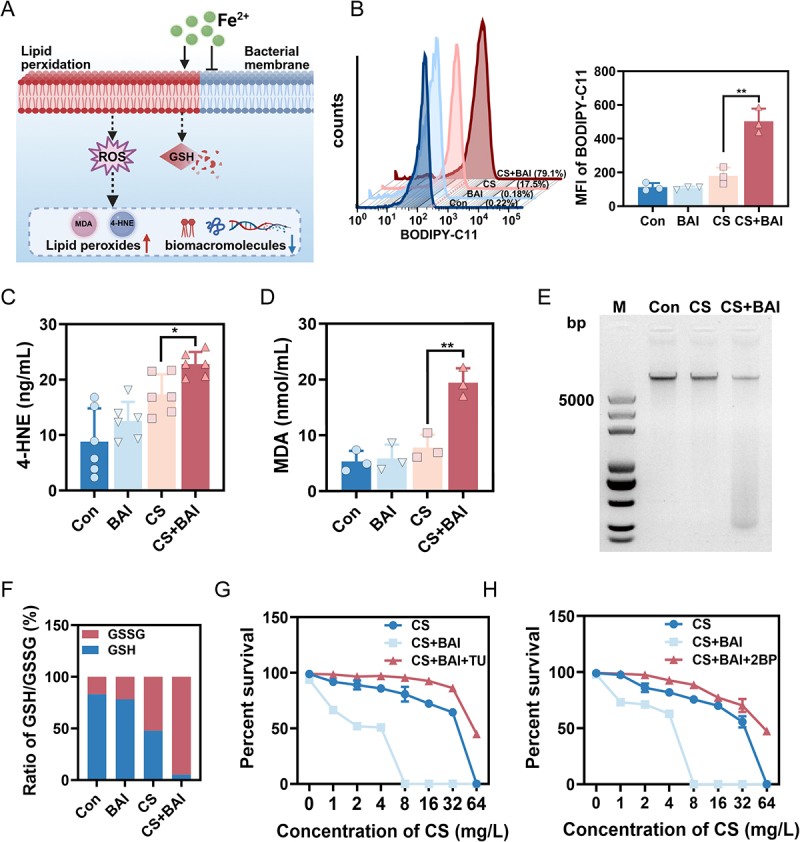
Iron homeostasis disruption by BAI augments CS killing by ferroptotic-like damage. (A) Scheme for ferroptotic-like damage generated by CS-induced reactive species in combination with BAI-induced ferrous iron accumulation; (B) Lipid peroxidation in bacterial cells treated by CS with or without BAI, as shown by the BODIPY-C11 probe (MFI: mean fluorescence intensity CS:1 μg mL^−1^, BAI: 25 μg mL^−1^); generation of typical ferroptotic-like byproducts, 4-HNE (C) and MDA (D) in bacteria treated by CS with or without BAI (CS:1 μg mL^−1^, BAI: 25 μg mL^−1^); (E) Genomic DNA degradation in bacteria treated by CS alone or CS-BAI combination (CS:1 μg mL^−1^, BAI: 25 μg mL^−1^); (F) Overproduction of RESs by the BAI-CS combination depletes the GSH in bacteria (CS:1 μg mL^−1^, BAI: 25 μg mL^−1^); (G) Scavenging reactive species by thiourea rescues bacteria under CS-BAI combination (TU: 100 mM); (H) Complexing ferrous iron by 2,2’-bipyridine rescues bacteria under CS-BAI combination (2BP: 0.25 mM) Data in B-H represented three biological replicates and were shown as mean ± SD. The unpaired *t*-test was used for the statistical analysis where * = *p* < 0.05, **= *p* < 0.01, *** = *p* < 0.001, **** = *p* < 0.0001. Schematics in panels A were created with BioRender.com (https://biorender.com).

Iron is an essential metal cofactor for biological processes in bacteria and an important component for normal intracellular reduction/oxidation reactions in living organisms [53]. Iron-associated redox reactions, through which oxidized species may be produced, are generally stringently balanced and governed. Once the iron overload occurs in a given condition, these processes are able to manipulate an iron-dependent form of cell death, which is termed ferroptosis in eukaryotes [54]. As BAI treatment led to an increase of ferrous iron at the expense of ferric iron, we wondered whether there would be biochemical consequences for BAI-mediated cellular iron dyshomeostasis in bacteria. Although ferroptosis has been rarely engaged for bacteria, both ROS and ferrous iron were found to be cumulated during BAI-CS treatment, suggesting the possible involvement of Fenton chemistry and ferroptotic-like damage therein (Figure 6A). Since lipid peroxidation catalyzed by excessive iron and ROS is a pro-ferroptotic hallmark in many living species, we first applied the BODIPY-C11 probe to detect the formation of lipid peroxide products in bacteria treated by CS and BAI-CS combination. As shown in Figure 6(B), compared with non-treated or CS alone-treated bacteria, the combination of BAI and CS triggered a significantly lipid peroxidation in bacteria. As a result of severe ferroptotic damage, 4-hydroxynonenal (4-HNE) and malondialdehyde (MDA), two common reactive metabolic byproducts from lipid peroxidation, were also identified to be significanly increased during combination treatment (Figure 6(C,D)). These two products are often generated from phospholipid or fatty acids on bacterial membranes via non-enzymatic lipid peroxidation, and thereafter attack the essential cellular components as highly reactive electrophilic species (RESs) [55]. This was confirmed by the genomic DNA of bacteria being degraded during combination treatment (Figure 6(E) & Fig. S13). Concurrently, massive productions of such RESs were also able to conjugate the glutathione (GSH) to deplete these reducing agents (Figure 6(F)), thus exacerbating the ferroptotic-like damage [56]. To finally validate the role of ferroptotic-like damage in BAI-mediated CS potentiation, thiourea (TU) was applied to quench the Fenton chemistry by scavenging the ROS. As expected, the addition of TU markedly increased the bacterial survival under the BAI-CS combination (Figure 6(G)). Consistently, application of 2,2’-bipyridine (2-BP) to complex the ferrous iron was also able to dampen the potentiated kill of CS in the presence of BAI (Figure 6(H)). These data together elucidated that BAI is able to potentiate the bactericidal activity of CS by inducing ferroptotic-like damage via dysregulating cellular labile iron.

### BAI enhances the CS efficacy in vivo

**Figure 7.**
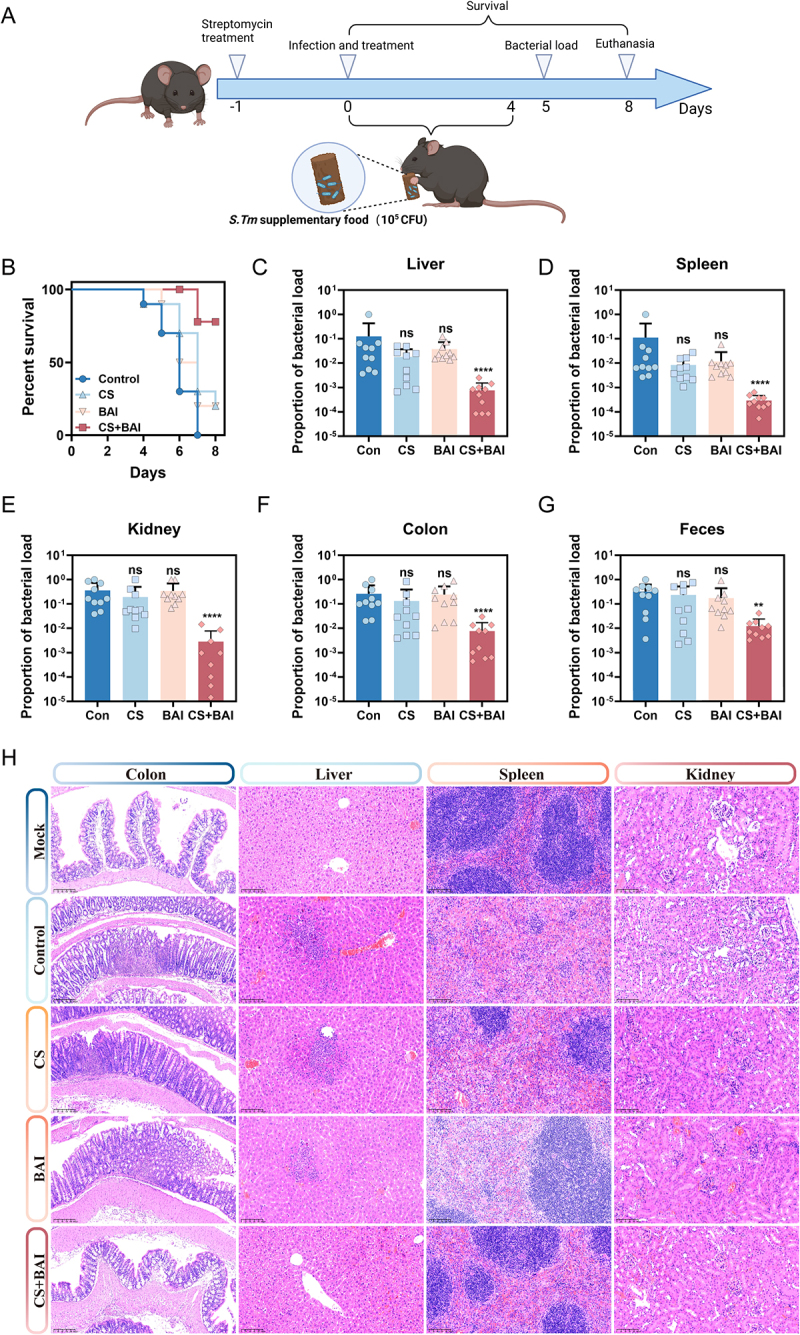
BAI-CS combination acts as a viable regimen *in vivo*. (A). Schematic illustration for the animal trial; (B). Survival of mice treated by BAI, CS and their combination after infection with *Salmonella* at a lethal dose (*Salmonella*: CFU g^−1^; BAI: 10 mg kg^−1^; CS: 5 mg kg^−1^); pathogen loads in liver (C), spleen (D), kidney (E), colon (F), and faeces (G) in mice treated by CS with or without BAI; (H). BAI, in combination with CS, alleviates the inflammation in the infected tissues. The magnification of the HE section is 20× . Data in B-H represented three biological replicates and were shown as mean ± SD. The Mann–Whitney test was used for the statistical analysis where * = *p* < 0.05, ** = *p* < 0.01, *** = *p* < 0.001, **** = *p* < 0.0001, ns not significant. Schematics in panels A were created with BioRender.com (https://biorender.com).

In light of promising synergism between BAI and CS *in vitro*, we should eventually evaluate the therapeutic efficacy of their combination *in vivo*. To this end, a murine infection model was established on the C57BL/6 mice that were pre-treated with streptomycin and then received *Salmonella*-containing (2 × 10^5^ CFU g^−1^) feed for 4 consecutive days. The infected mice were then treated with PBS (control), CS, BAI, and their combination (Figure 7(A)), and the survival rate, bacterial load, and histopathology of treated mice were determined. The results showed that the survival rate of mice that received BAI-CS combination therapy significantly increased to 77.8%, which was much higher than that treated by BAI (20%) or CS (20%) alone (Figure 7(B)). The bacterial loads of infected mice were measured at 5 d post-intervention. The high bacterial burdens were detected in the liver, spleen, kidney, colon, and feces from mice that received different treatments. The applications of BAI or CS negligibly affected the pathogens in such tissues, whereas the BAI-CS combination resulted in a significant reduction in the bacterial burden by approximately 10^2^-10^4^ CFU compared to the treatment of each alone (Figure 7(C-G)). The therapeutic outcome of the BAI-CS combination was further affirmed by histological analysis. As shown in Figure 7(H), the *Salmonella* infection induced significant histopathological damage in multiple tissues. In the intestine, infection resulted in a marked reduction of goblet cells in the intestinal mucosa, accompanied by diffuse inflammatory cell infiltration extending into the lamina propria. In the liver, extensive and diffuse inflammatory cell infiltration was observed, leading to necrotic lesions with mild fibrosis. The spleen exhibited dilated sinusoids, thickened trabeculae, and increased disorganized fibrous tissue. Additionally, diffuse neutrophil infiltration was evident, along with indistinct germinal centers and disrupted architecture of the red and white pulp. However, the treatments by BAI or CS alone only partially alleviated the tissue damage and inflammation that occurred due to the *Salmonella* infection. Remarkably, combinatorial therapy with BAI-CS almost completely abolished the inflammatory damage as observed. Beyond its promising therapeutic efficacy, the combination of BAI and CS also exhibited encouraging *in vivo* safety, as no obvious abnormalities were observed in the histopathological analysis (Fig. S14). It was, therefore, concluded that BAI potentiates the CS activity for better rescuing the infection *in vivo*, highlighting the potential of the BAI-CS combination as a viable therapeutic regimen to combat the pathogens of clinical importance.

## Discussion

The antimicrobial regimen based on the traditional AST was severely reported to fail in the clearance of infections caused by *Salmonella* or *Enterobacter cloacae* in murine models due to transient antibiotic resistance induced by host conditions [57,58]. This behavior, termed *in vivo* altered susceptibility (IVAS) by Ersoy and colleagues, not only elicits failure in predicting accurate dosing protocol but also accounts for great bias that may omit many *in vivo* effective agents in drug discovery [30]. There are several reasons behind this behavior. First, the defined host and *in vivo* conditions often impose nutrient limitations, which may alter bacterial susceptibilities and responses upon exposure to certain molecules [59]. Second, the different ion strengths in such conditions vs the supra-physiologic levels in standard CAMH contribute to regulating the membrane physiology and efflux activity of bacteria that are able to cooperatively modulate the accumulation of certain molecules [60,61]. Third, the infection sites generally offer a mildly acidified and oxidative microenvironment, which may directly induce bacterial stress responses to manipulate the efficacies of antibiotics and antibiotic adjuvants [23]. Our findings in this study align closely with these observations, as the CS exhibited significantly diminished activity (4–64-fold MIC) in our selected medium than that in the standard CAMH. This divergence in MIC has direct translational implications for sporadically reported treatment failure of CS at routinely used doses and highlights a necessity for effective adjuvants to add a treatment option at a time when CS therapies are limited. To this end, we exploited a modified screening procedure that assembled the host milieu to observe the interaction between CS with chemicals in stock and identified BAI as the most potent lead to potentiate CS. There are several host-relevant media that have been applied to illustrate how bacteria respond to antimicrobials or stressors, e.g. the DMEM, RPMI, nose-mimicking medium, lung-mimicking medium, blood-mimicking medium, and macrophage-mimicking medium [62–64]. In the present study, the rationale for selecting macrophage-mimicking LPM is that successful residence and replication in such phagocytes are the precondition for *Salmonella* to establish systemic infection [65]. As such, the *Salmonella* are able to subvert the host antimicrobial stress and simultaneously evade the antibiotic eradications by transient adaptive mechanisms [66]. Thus, despite the current work holding certain limitations in recapitulating host aspects like serum protein or immune responses, elucidating the antibiotic adjuvants with niche-specific activity at the phagocytic milieu is still of primary interest and translationally pronounced to preclude inadvertent exclusion of molecules active against *Salmonella in vivo*.

Although the resistance to CS can be gained by complete loss of LPS biosynthesis via mutations on the lipopolysaccharide peroxidation (LpX) pathway, this mechanism has only been observed in *A. baumannii* and has been identified to be lethal for other organisms [67]. Therefore, in general, the Gram-negative germs typically exploit LPS modifications to reduce the affinity of CS to the bacterial membrane. The emergence of *mcr* genes or their variants on mobilized plasmids in 2015 has soon been on an upstaging of other resistant mechanisms, since their trait in plasmid-mediated transfer menaces CS as the backbone of regimens against multiply resistant Gram-negative bacilli [68]. While *mcr* genes have been consistently reported to only induce low-level resistance across species [69]. And overexpressing *mcr* to promote the CS resistance is not possible since *mcr* genes incur a severe fitness cost on the host bacterium and compromise bacterial viability [70]. Thus, the adaptive CS resistance by chromosomally mediated modulation of two-component regulatory systems has been reinvigorated in clinical importance recently, as these modes of action are able to confer high-level resistance at rather low cost [71,72]. This is in consistence with the observation in this study that bearing *mcr*-1 plasmid only demonstrated moderate resistance comparing to their counterparts (2–8 μg mL^−1^), whilst the adaptive mechanism that adaptively primed by two-component system in LPM directly led to higher levels CS resistance (>64 μg mL^−1^), and it acted synergistically with mutations in *mgrB* to further augment the resistance (up to 256 μg mL^−1^). Interestingly, BAI identified in the current work was found to specifically target the aforementioned response to lessen the LPS modification through activation of PmrA/B. This BAI-induced PmrA/B deactivation acted on both CS-sensitive and -resistant isolates, implying the adaptive mediators (i.e. PmrA/B, PhoP/Q, or MgrB) of CS resistance are promising target treatment paradigms since their inhibition can either restore or potentiate the CS activity where it applies. Notably, in our study, deletion of *pmrA/B* completely abolished the synergistic activity between BAI and CS, whereas deletion of *phoP/Q* did not. This finding suggests that PmrA/B is the primary regulatory determinant of the downstream LPS modifications associated with CS susceptibility, while PhoP/Q functions mainly as an auxiliary module whose action is highly PmrA/B-dependent. Meanwhile, PmrAB regulatory network may be wired differently across Gram-negative bacteria and the magnitudes of its biological consequence may differ among species. Therefore, although these two systems are functionally linked, our results indicate that intact PmrA/B signaling is more critical for the development of CS potentiators.

**Figure 8.**
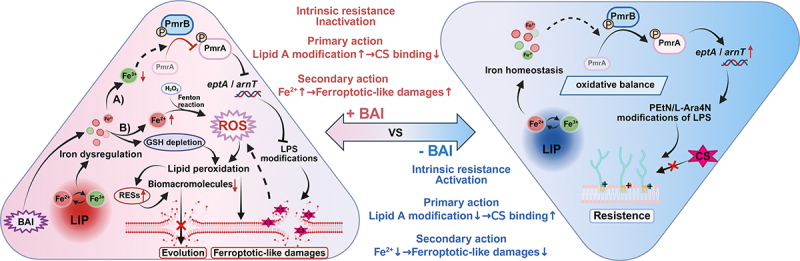
Model of cellular labile iron pool dictating bacterial susceptibility to CS. (A). Reduction in ferric and total iron shuts the PmrA/B signaling to enhance CS binding and destabilize the bacterial membrane; (B). Increment of ferrous iron responds to CS-dependent ROS to generate lethal ferroptotic-like damages for better pathogen eradication. Schematics were created with BioRender.com (https://BioRender.com).

Due to the clinical significance of CS as a last resort antibiotic, many preceding investigations made great attempts to elaborate CS adjuvants or synergists to replenish its clinical utility. However, prior efforts to overcome CS resistance have placed large emphasis on nonspecific membrane disruptors or efflux pump inhibitors, but these approaches lack mechanistic selectivity and may aggravate CS-induced toxicity [73]. Hence, an in-depth illustration of genetically dissected MOA and molecular targets therein is favored to aid in the design of improved next-generation combinatorial therapy. A good example is the pioneering study by Carfrae and coworkers, where the fatty acid synthesis (FAS) inhibitors (MAC13772, etc.) were found to sensitize MDR pathogens to CS. This work not only proposed several leads for restoring CS but also shed light on the intriguing association between FAS and CS resistance, which can be an optimal target to develop state-of-the-art compounds as viable adjuvants [15]. In this work, we revealed that the labile iron is an important cue to be exploited as a promising strategy to potentiate CS efficacy *in vivo*. As one of the abundant metal substances, the amount and form of iron are strictly fine-tuned in bacterial cells for iron homeostasis. In the presence of an iron disruptor, for instance, BAI as proof-of-concept in this study, the ferrous iron soon presents as dominant at the expense of depletion of ferric and total iron. This reshaped labile iron pool functions as a nexus linking the primary action of CS to bind the negatively charged LPS and its subsequent secondary action to induce oxidative stress [74]. For the primary action, depletion of ferric iron indirectly blocks the PmrA/B-dependent membrane modifications, which increases the CS affinity to LPS to damage the bacterial membrane integrity (Figure 8(A)). On the other hand, the accumulation of ferrous iron responds to generated ROS to mediate the ferroptotic-like damage that deteriorates the cellular components for better bactericidal activity (Figure 8(B)). Although we have reported that modulation of iron was able to manipulate the CS binding in bacteria in our prior investigation [21], this work is considered to be the first attempt, to our best knowledge, to illustrate that the ferroptotic-like damage is the collateral biological consequence of labile iron pool perturbation and CS treatment. It is also notable that the CS in combination with the iron disruptor manifests a lower possibility of the development of spontaneous resistance. This is controversial at first glance since the generation of ROS has been deemed an incubator for de novo acquisition of resistance [75]. We assume that the ferroptotic-like damage dictated by iron dysregulation could be the driver for diminished evolution. As probed in this study, RES have been produced as the byproducts of the ferroptotic-like damage. These reactive byproducts induce DNA breakdown instead of lesions, consequently draining the necessary components for evolution. Thus, the labile iron pool disruption may play a multifaceted role in treatment paradigms based on CS.

## Conclusion

To conclude, we demonstrated that cellular labile iron serves as a determinant of CS susceptibility in bacteria under host conditions or *in vivo*. Manipulating iron homeostasis by disruptors, such as BAI in this study, is able to potentiate the CS activity and prevent the resistance development through modulating PmrA/B-dependent membrane modifications and ferroptotic-like damages. The present study not only provides a translationally viable regimen against bacterial infection in the clinic but also unveils an exploitable association between cellular labile iron with CS susceptibility, which may inspire the combination therapies targeting the hard-to-treat Gram-negative infections.

## Supplementary Material

SI to MS QVIR 2025 1048.docx

## Data Availability

The datasets were generated or analyzed during this study are deposited in Figshare (http://doi.org/10.6084/m9.figshare.30958295).
