# Supplementary material for: Natural molecule potentiates colistin efficacy in vivo via modulating adaptive LPS modifications and ferroptotic-like damages
Source: Virulence. 2026 Jul 21;17(1):2707759. doi: 10.1080/21505594.2026.2707759 (PMC13418482; doi:10.1080/21505594.2026.2707759)
Supplement: SI to MS QVIR 2025 1048.docx [file KVIR_A_2707759_SM4946.docx]

Supporting Information

Natural molecule potentiates colistin efficacy *in vivo* via modulating adaptive LPS modifications and ferroptotic-like damages

Hui-Hui Zhang ^a,b,c^, Yi-Dan Cao ^a,b,c^, Ying-Ying Xie ^a,b,c^, Yu-Ze Li ^a,b,c^, Xiao-Na Fan ^a,b,c^, Qiu-Yue Diao ^a,b,c^, Yu-Jiao Liang ^a,b,c^, Li-Ren He ^a,b,c^, Zi-Xing Zhong ^a,b,c^, Li-Juan Xia ^a,b,c^, Ze-Hua Cui ^a,b,c^, Xiao-Ping Liao ^a,b,c^, Xin-Lei Lian ^a,b,c^, Dong-hao Zhao ^a,b,c^, Jian Sun ^a,b,c,*^, Hao Ren ^a,b,c,*^

1. State Key Laboratory of Animal Disease Control and Prevention, College of Veterinary Medicine, South China Agricultural University, Guangzhou, China
2. National Risk Assessment Laboratory for Antimicrobial Resistance of Animal Original Bacteria, South China Agricultural University, Guangzhou, China
3. Guangdong Provincial Key Laboratory of Veterinary Pharmaceutics Development and Safety Evaluation, South China Agricultural University, Guangzhou, China

**^*^** Correspondence to: 483 Wushan Street, Tianhe District, Guangzhou 510642, China

*E-mail addresses:* [hao.ren@scau.edu.cn](mailto:hao.ren@scau.edu.cn) (H. Ren); [jiansun@scau.edu.cn](mailto:jiansun@scau.edu.cn) (J. Sun).


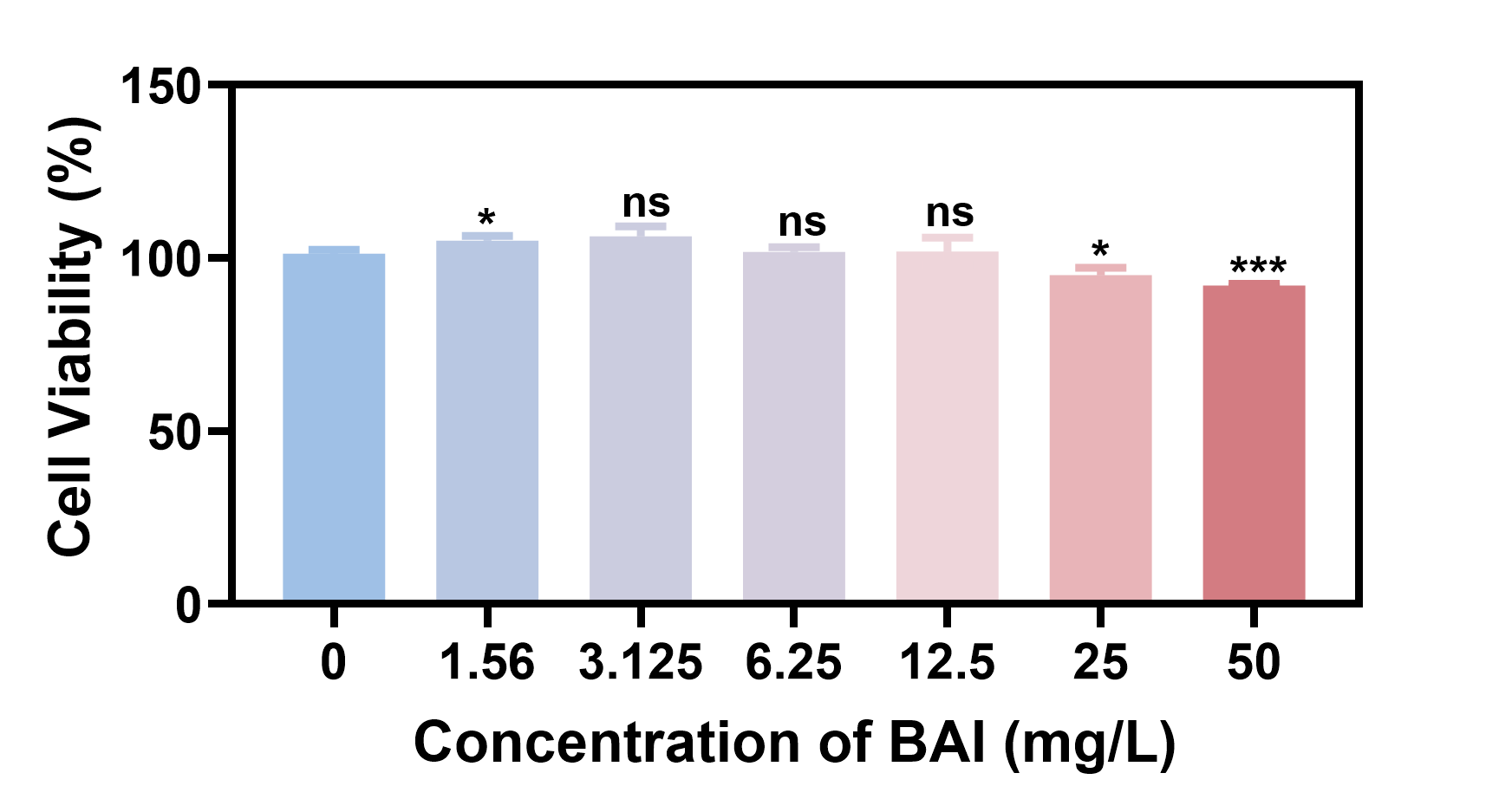


**Figure S1.** Cell viability of RAW264.7 induced by BAI after 12 h in CCK-8 assay. The unpaired *t*-test was used for the statistical analysis where *=*p*<0.05, ***= *p* <0.001, ns not significant.


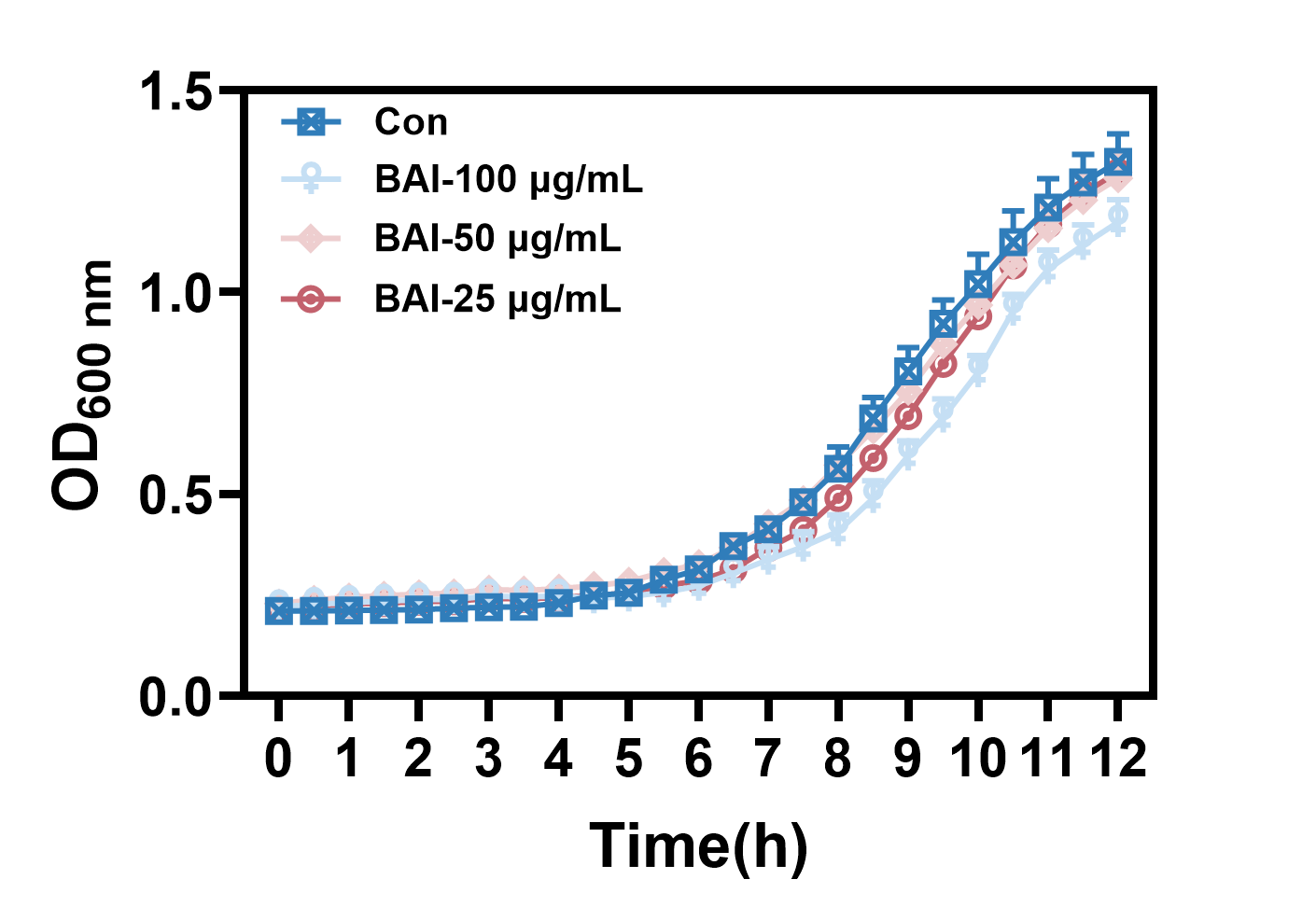


**Figure S2.** Effect of different concentrations of BAI on the growth of *Salmonella*. *Salmonella* was cultured in the presence of 0-100 µg mL^-1^ BAI, and bacterial growth was monitored at various time points. The results indicated that BAI, at the tested concentrations, did not significantly inhibit the growth of *Salmonella*.

**
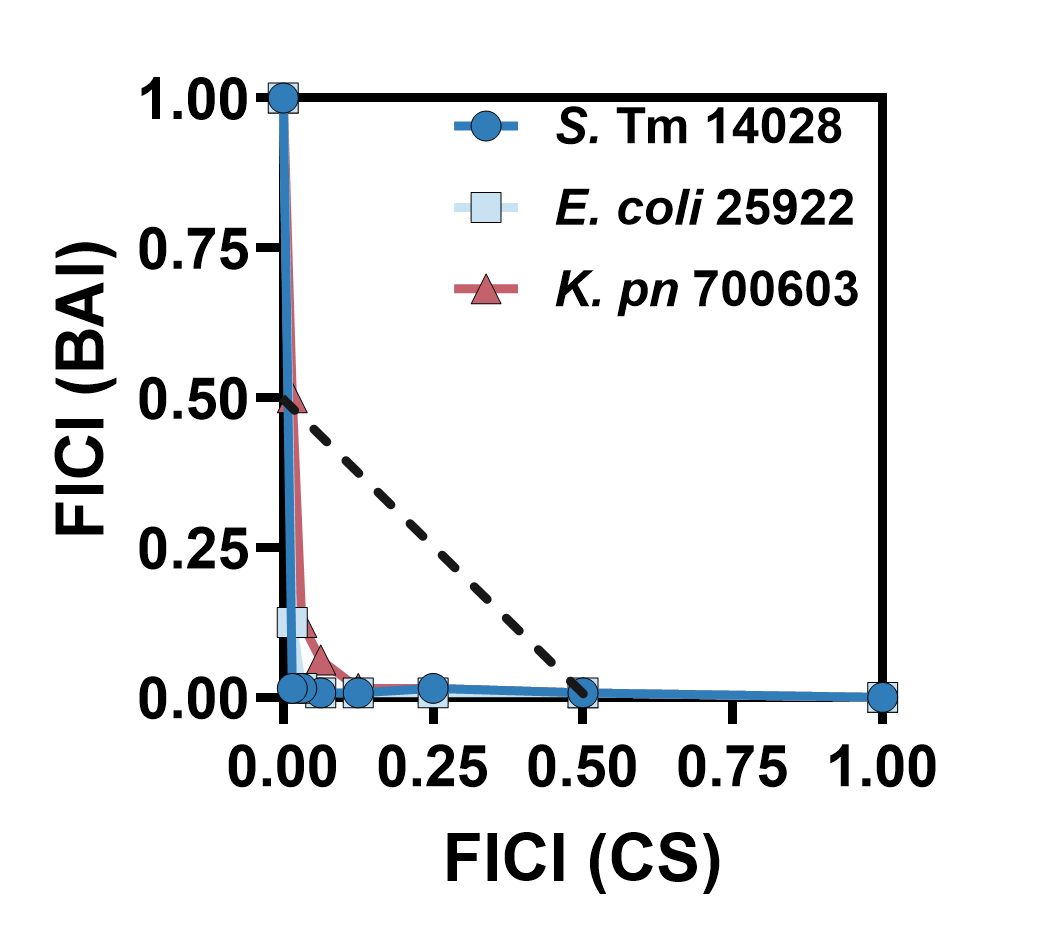
Figure S3.** Isobolograms of the combination of colistin and BAI against different Gram-negative bacterial strains. Strains of tests, including *S*. Typhimurium: ATCC 14028; *E. coli*: ATCC 25922; *K. pneumoniae*: ATCC 700603.


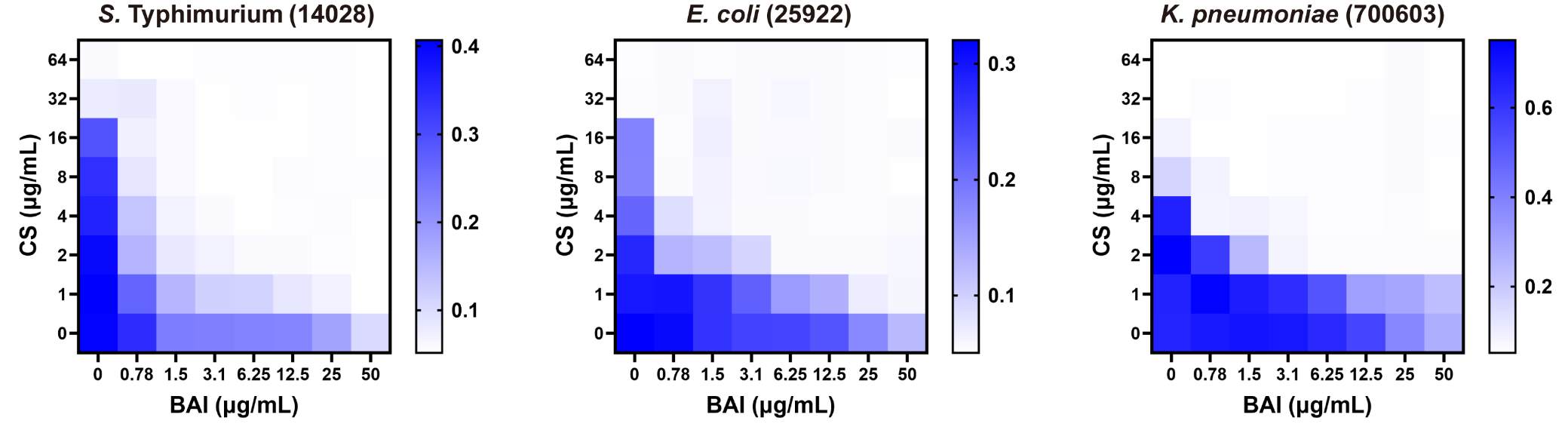


**Figure** **S4.** Checkerboard assays evaluating the combination of colistin and BAI against different Gram-negative bacterial strains. Blue color intensity is proportional to bacterial growth density, with darker blue indicating higher growth. Strains of tests including *S*. Typhimurium: ATCC 14028; *E. coli*: ATCC 25922; *K.pneumoniae*: ATCC 700603.


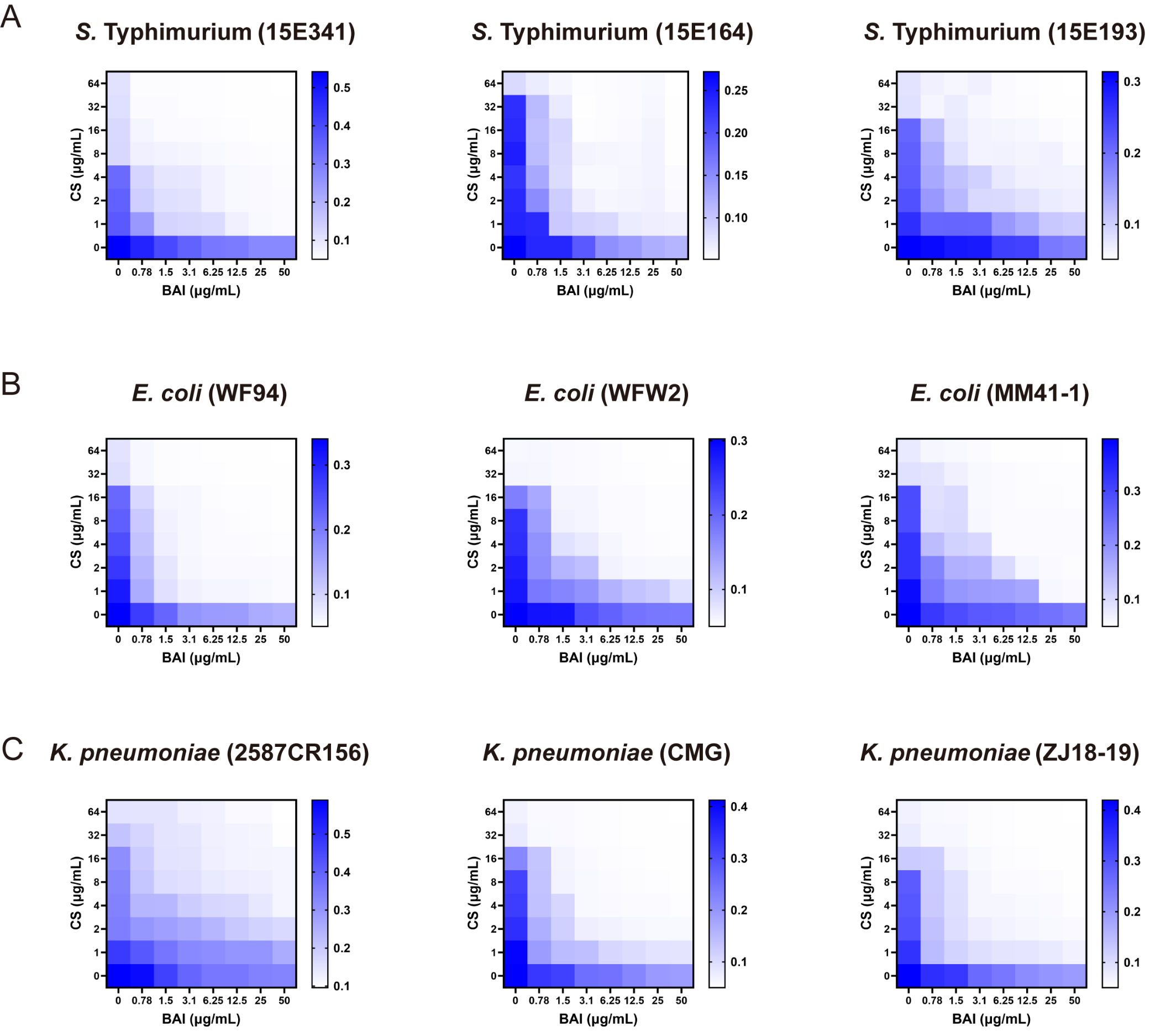


**Figure S5.** Checkerboard assays evaluating the combination of colistin and BAI against different colistin-resistant Gram-negative bacterial strains. Blue color intensity is proportional to bacterial growth density, with darker blue indicating higher growth. Strains of tests including *S.* Typhimurium: 15E341, 15E164, 15E193 (A); *E. coli*: WF94, WFW2, MM41-1 (B); *K. pneumoniae*: CMG, ZJ18-19, 2587CR156 (C).


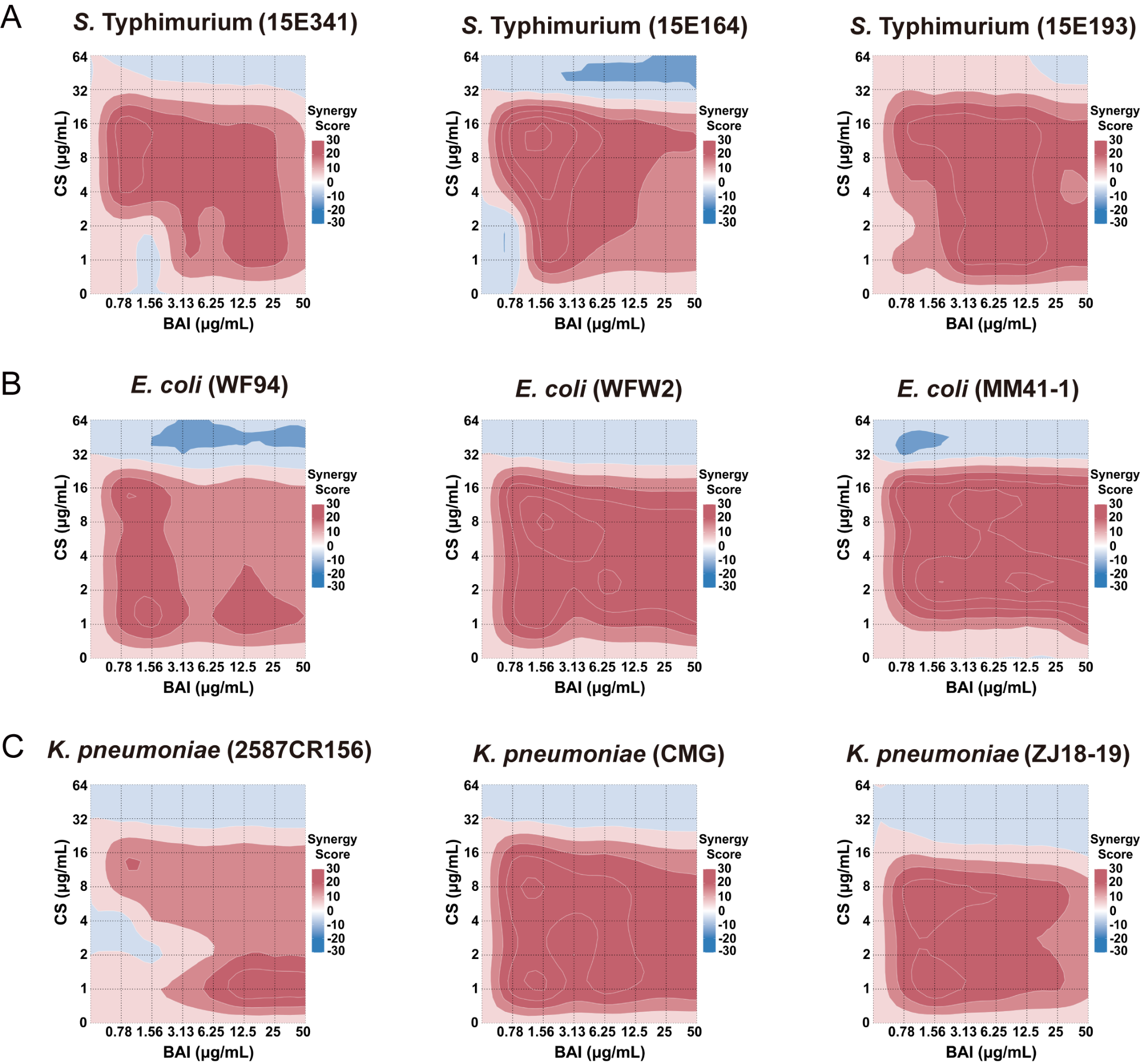


**Figure S6.** The synergism landscape of BAI and colistin on colistin-resistant isolates (the combination doses in the red area indicated a synergistic response, while those in the blue area indicated indifference). The experiments were performed on the isolates of *S.* Typhimurium (A), *E. coli* (B), and *K. pneumoniae* (C).

**
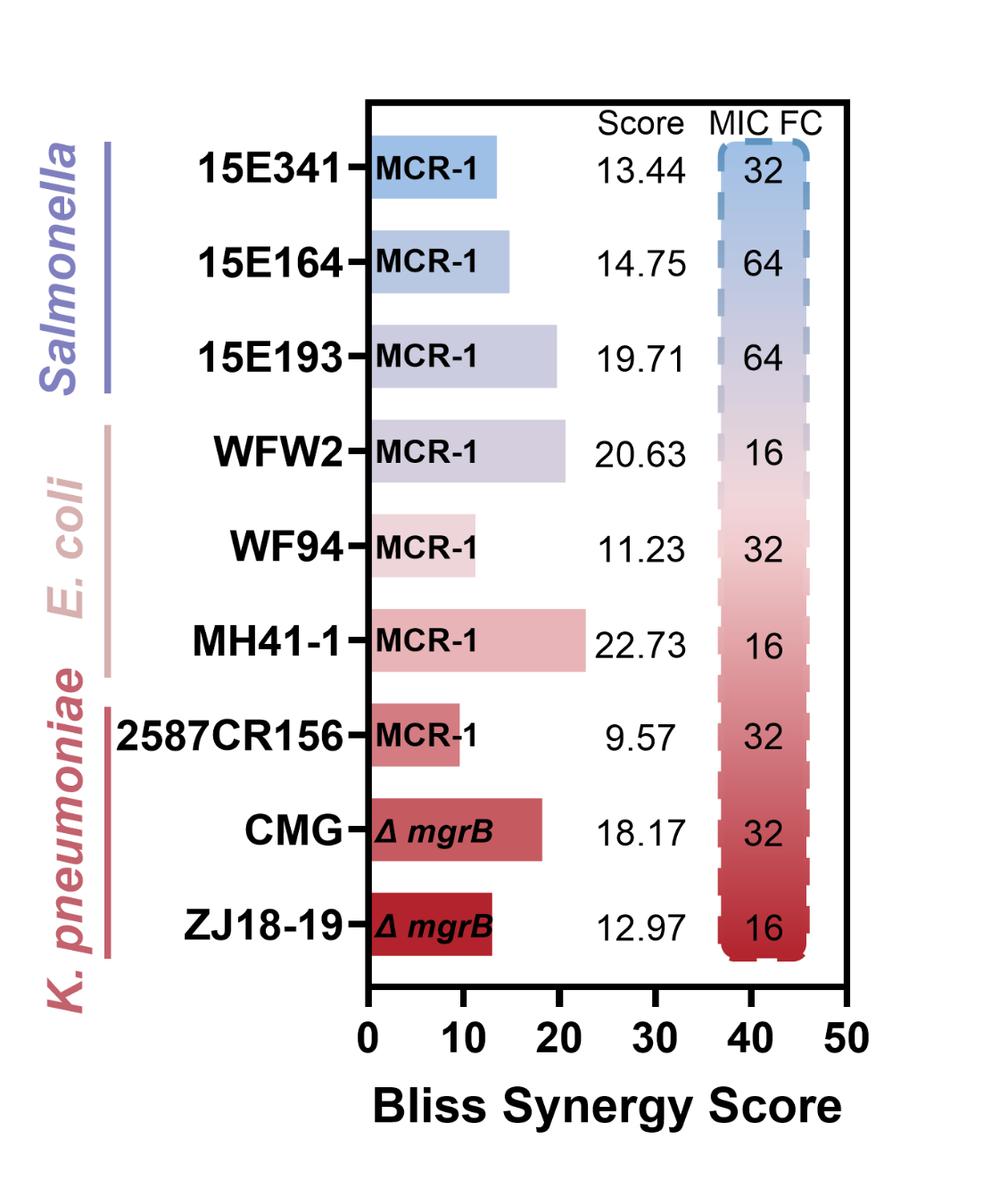
Figure S7.** BAI restores colistin activity against different colistin-resistant isolates harboring *mcr* allele or *mgrB* mutations. The Bliss score of colistin-BAI combination on colistin-resistant isolates; FC = fold change.


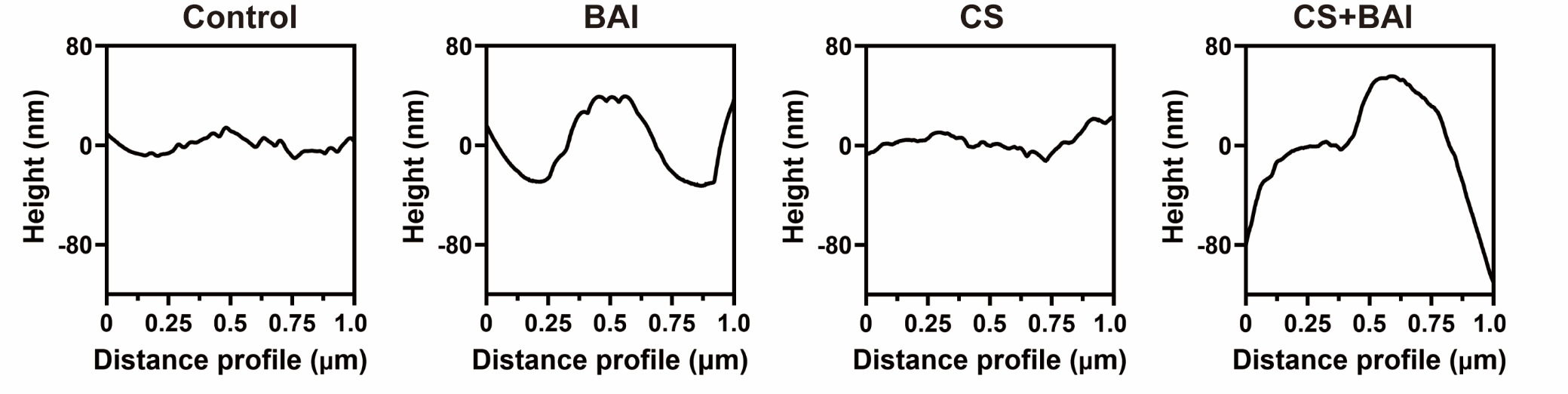


**Figure S8.** The membrane roughness was detected by Atomic force microscopy (AFM) after treatments of colistin (1 μg mL^-1^) or in combination with BAI (25 μg mL^-1^).


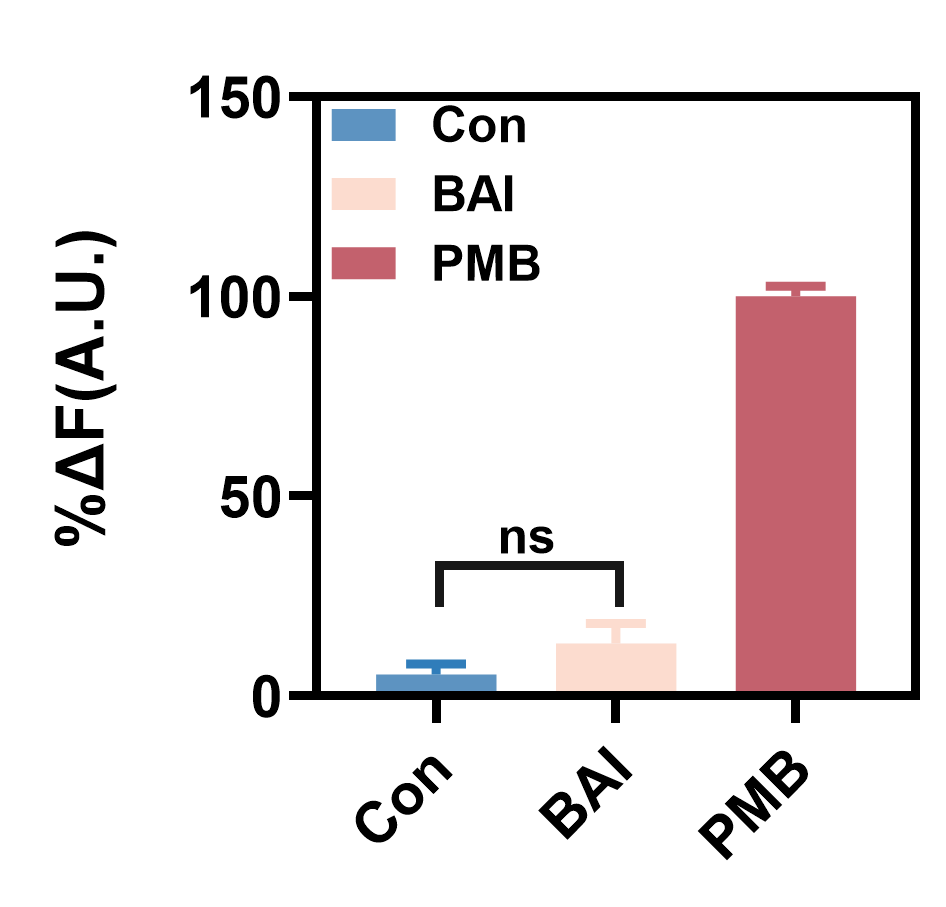


**Figure S9.** LPS binding ability of BAI (3.125 μg mL^-1^) to LPS from *S.* Typhimurium, with 10 μg mL^-1^ polymyxin B treatment as a positive control.


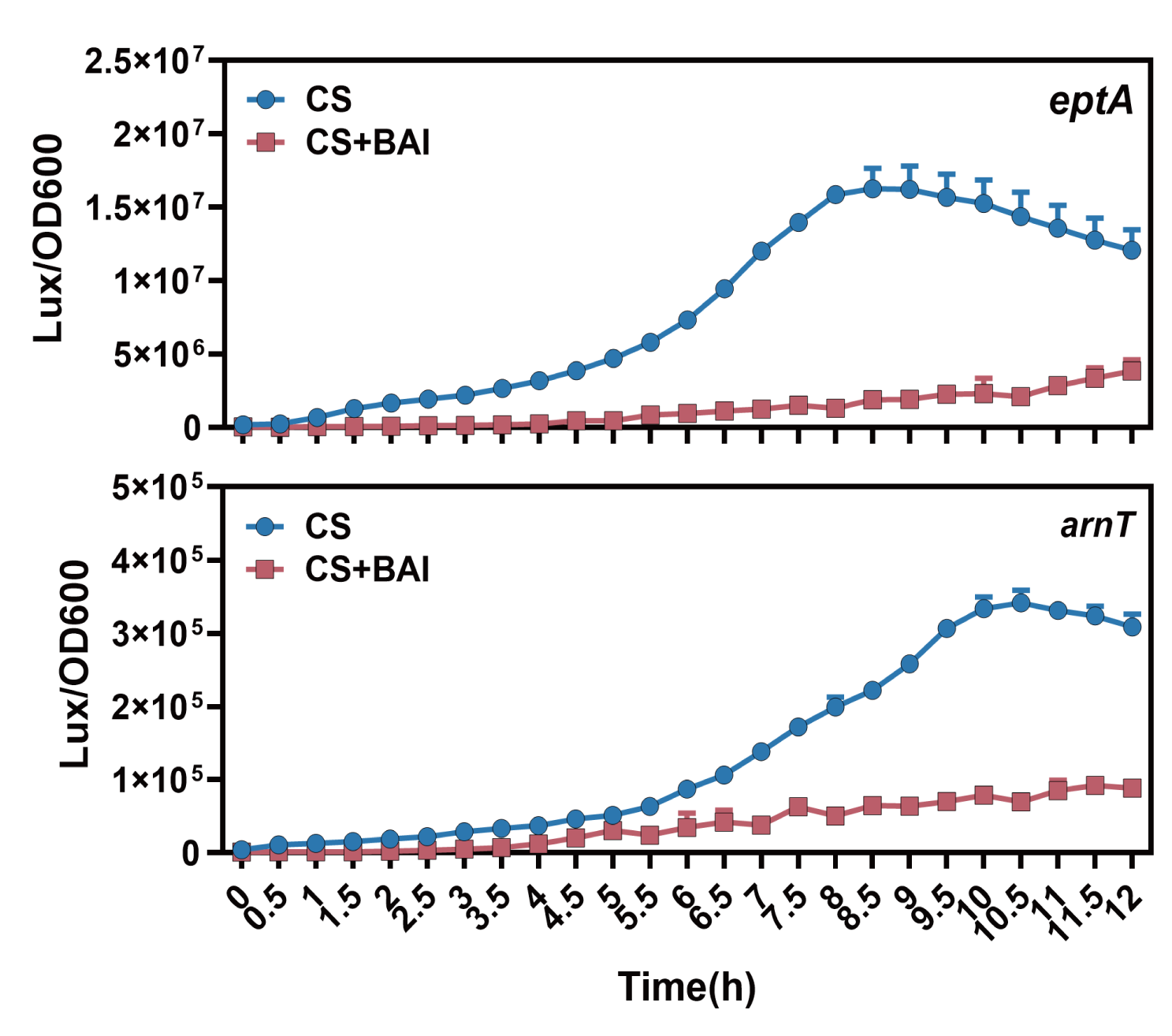


**Figure S10.** Expression dynamics of *eptA* and *arnT* in the presence or absence of BAI.


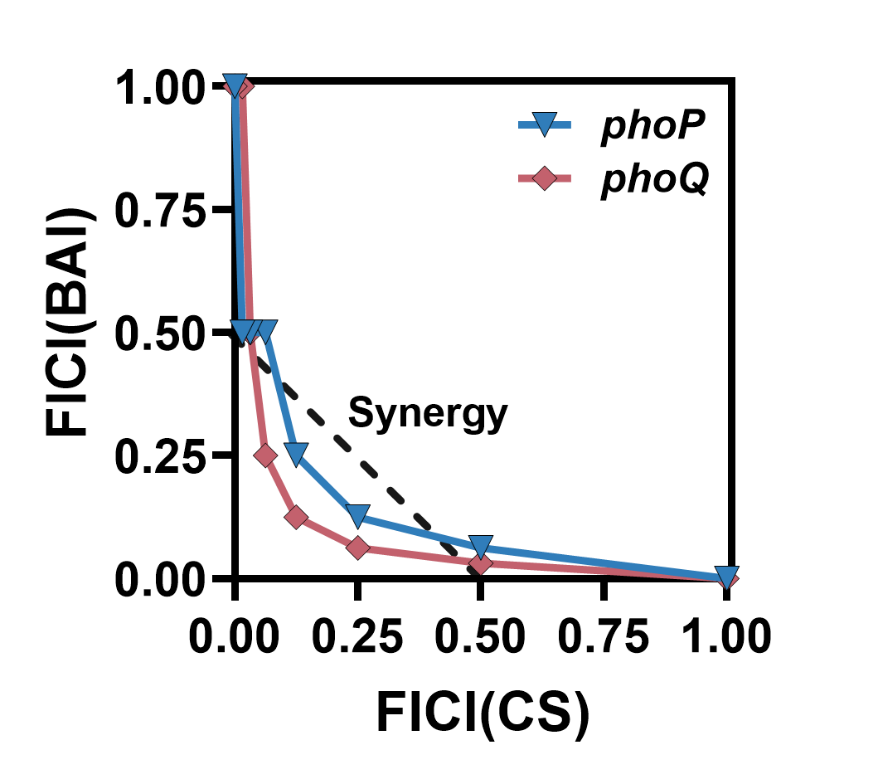
**Figure S11.** Absence of PhoP/Q system only partially dampens the colistin potentiation by BAI.


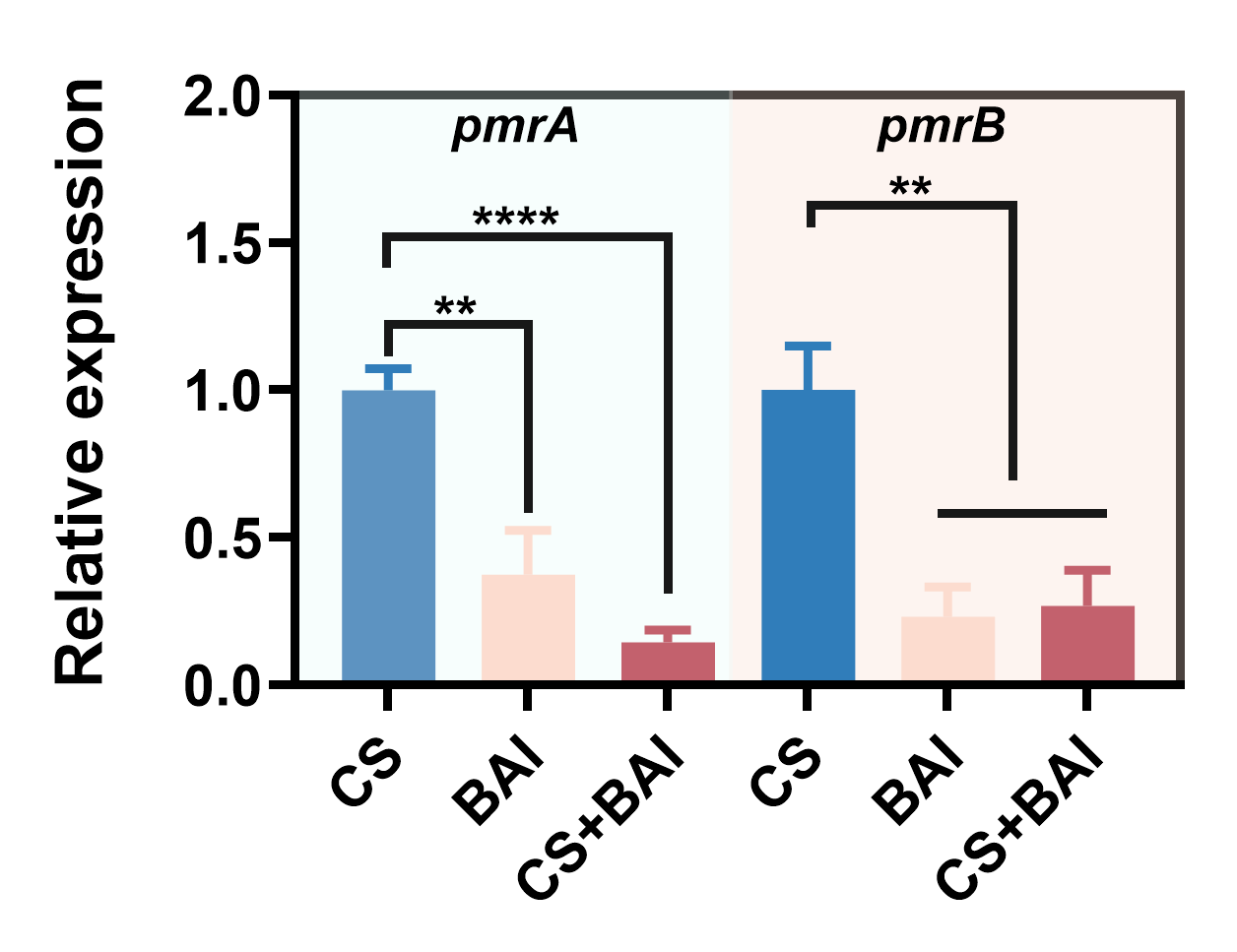


**Figure S12.** BAI (25 μg mL^-1^) and its combination with CS (1 μg mL^-1^) spontaneously reduce the expression of *pmrA* and *pmrB*. The unpaired *t*-test was used for the statistical analysis where *=*p*<0.05, **= *p* <0.01, ***= *p* <0.001, ****= *p* <0.0001, ns not significant.


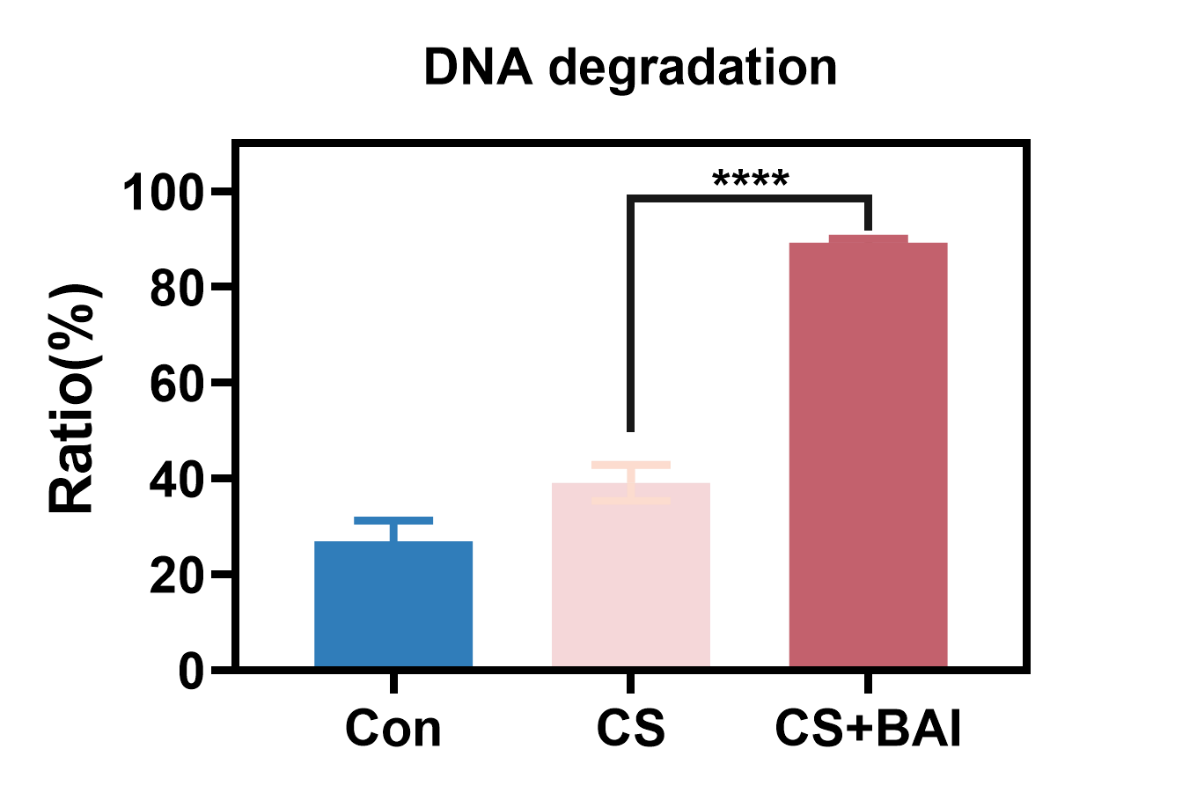
**Figure S13.** BAI-colistin combination (CS:1 μg mL^-1^, BAI: 25 μg mL^-1^) generates RESs to degrade the genomic DNA. The unpaired *t*-test was used for the statistical analysis, where ****= *p* <0.0001, ns not significant.


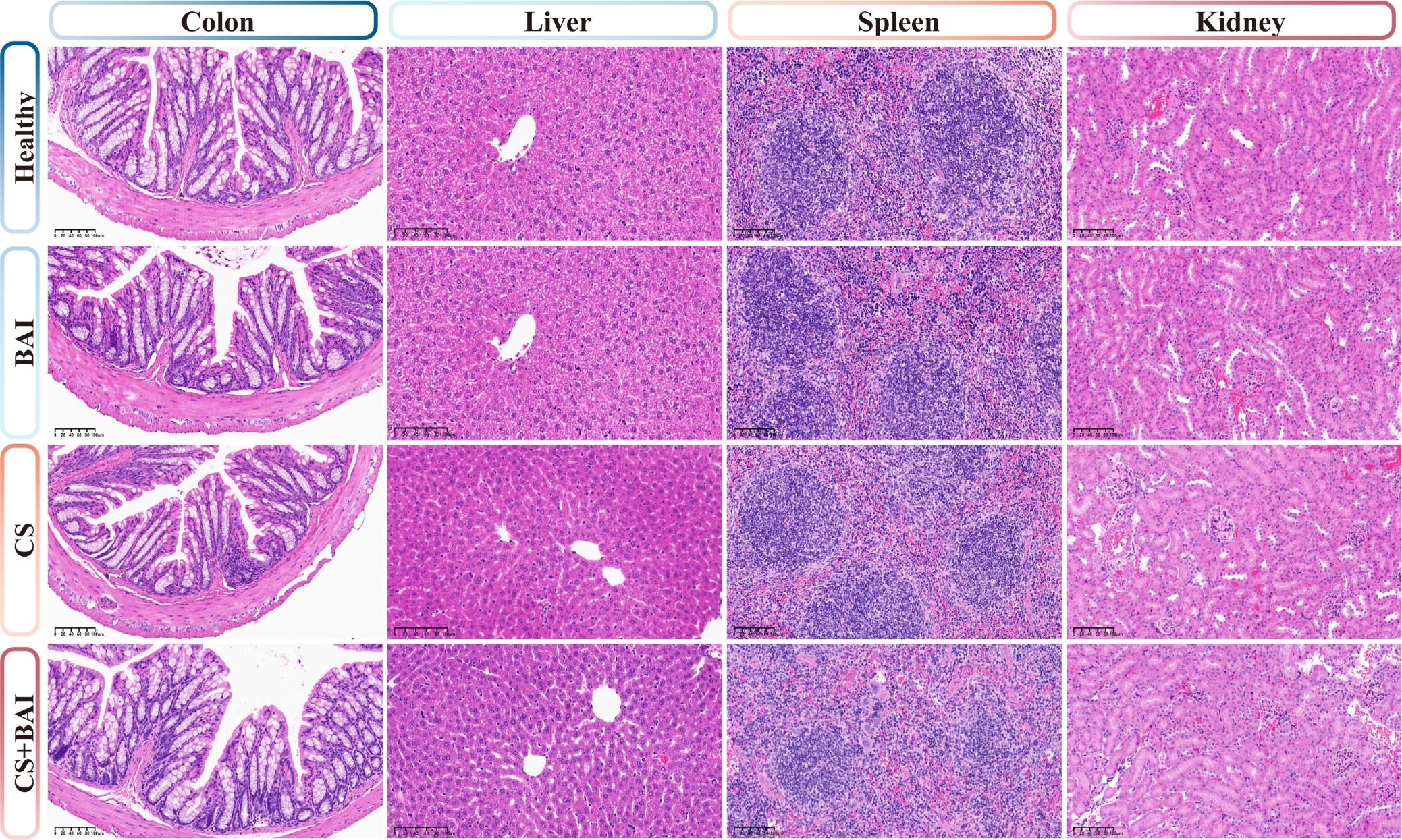


**Figure S14.** The biosafety assessment of CS (5 mg kg^-1^), BAI (10 mg kg^-1^), and their combination *in vivo*. The magnification of HE of the liver was 20×, and the magnification of HE sections of the colon, kidney, and spleen was 15×.

**Table S1**

Phytochemicals used in the primary screening

| **Phytochemicals** | **ε̃-value** | **Structure** |
| --- | --- | --- |
| Verbenalin | 0.951811 | 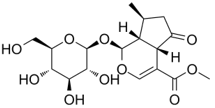 |
| Polyphyllin VI | 0.873427 | **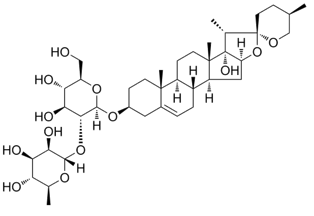** |
| Saikosaponin B2 | 0.785381 | **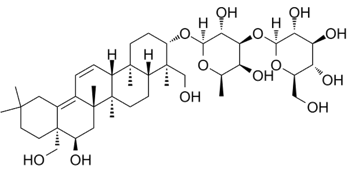** |
| Platycodin D | 0.967434 | **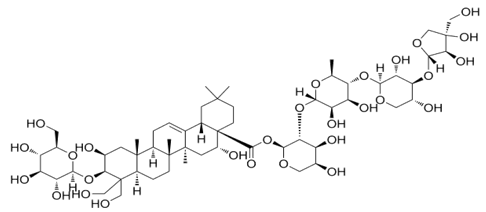** |
| Toosendanin | 0.596671 | **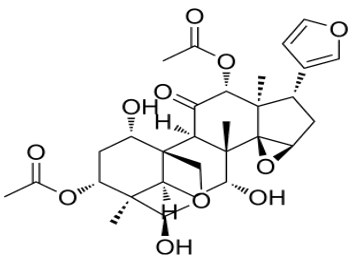** |
| Corynoxeine | 0.569214 | **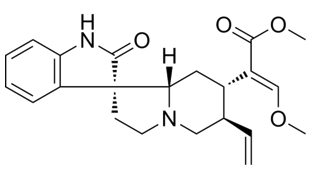** |
| Shanzhiside methyl ester | 0.499111 | 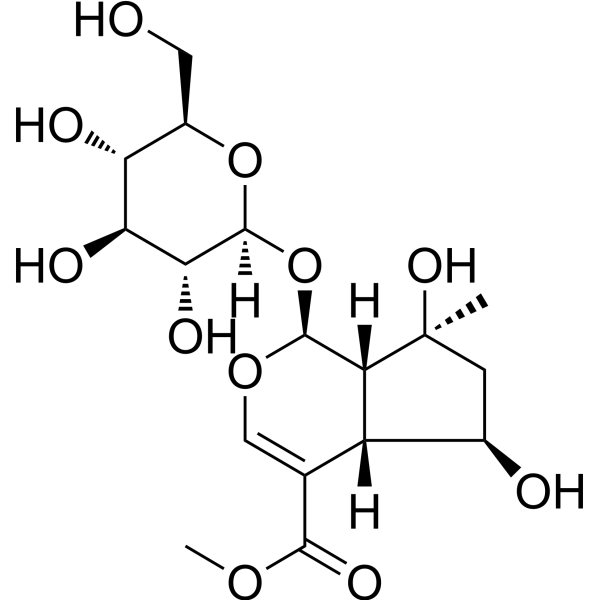 |
| Pulsatilla saponin D | 0.48841 | 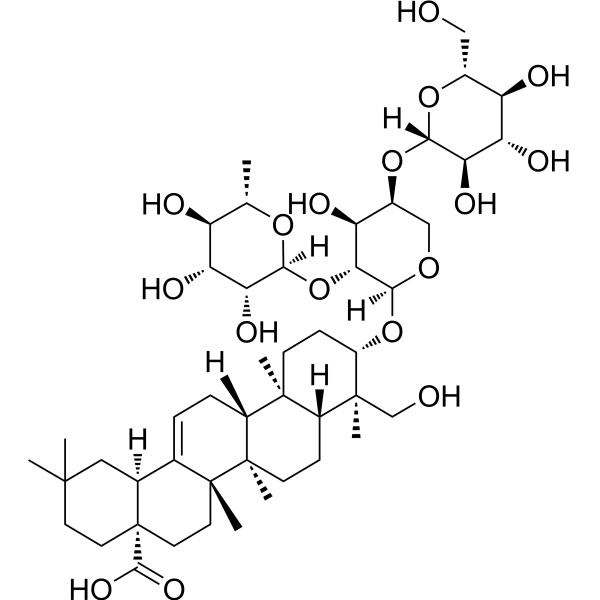 |

**Table S1**

Phytochemicals used in the primary screening (continued)

| **Phytochemicals** | **ε̃-value** | **Structure** |
| --- | --- | --- |
| Micheliolide | 0.913263 | 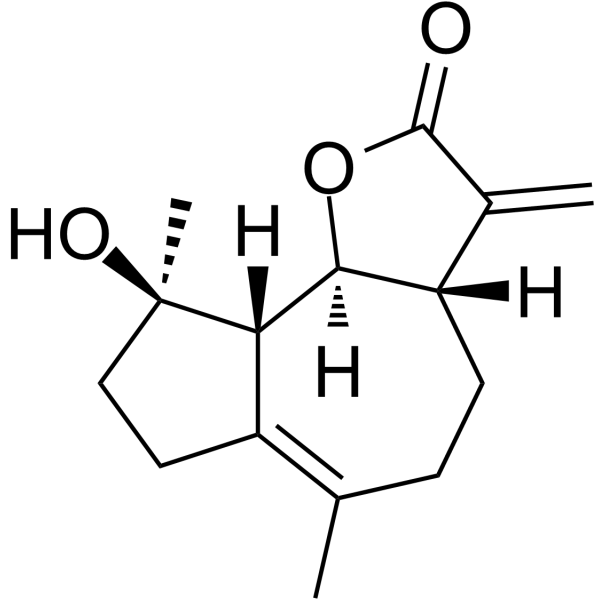 |
| Isorhychophylline | 0.858154 | **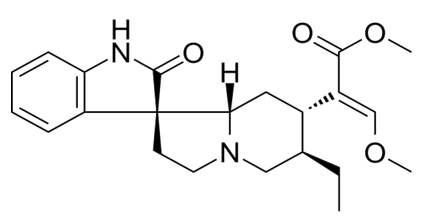** |
| Germacrone | 0.930197 | 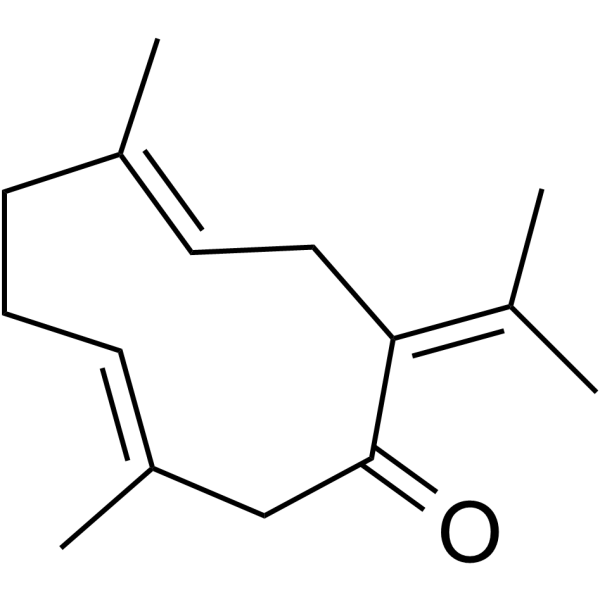 |
| Cyanidin-3-O-glucoside chloride | 0.883782 | 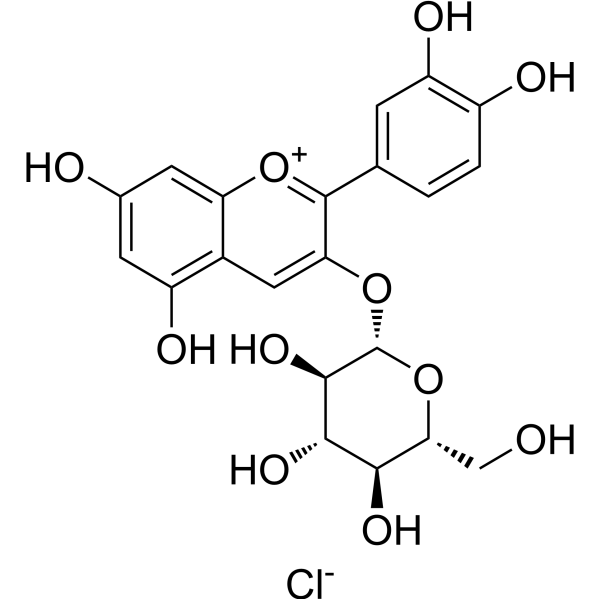 |
| Atractylenolide III | 0.813253 | **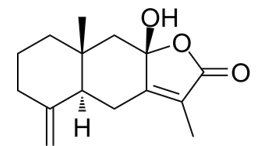** |
| Atractylenolide II | 0.565629 | **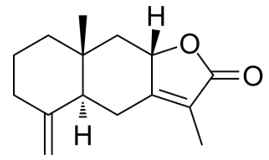** |
| Praeruptorin A | 0.474898 | **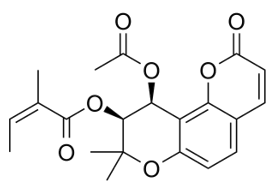** |
| Polyphyllin II | 0.552434 | **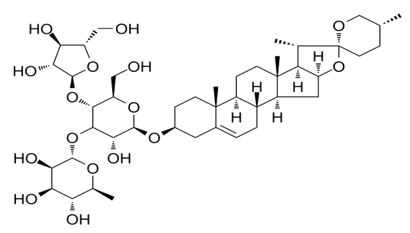** |

**Table S1**

Phytochemicals used in the primary screening (continued)

| **Phytochemicals** | **ε̃-value** | **Structure** |
| --- | --- | --- |
| Forsythoside A | 0.601678 | **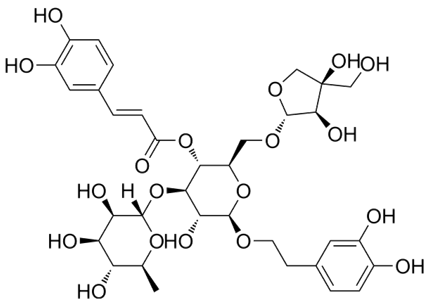** |
| (20R)-Ginsenoside Rh1 | 0.659649 | **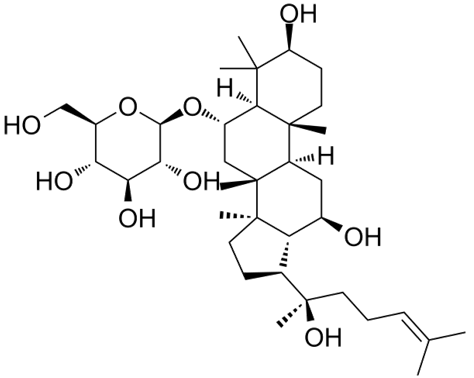** |
| Cryptochlorogenic acid | 0.637909 | **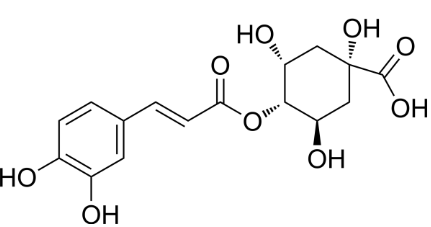** |
| Efetaal | 0.669455 | 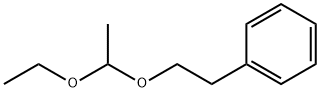 |
| Topotecan | 0.649626 | **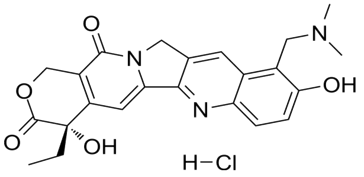** |
| Neodiosmin | 0.59422 | 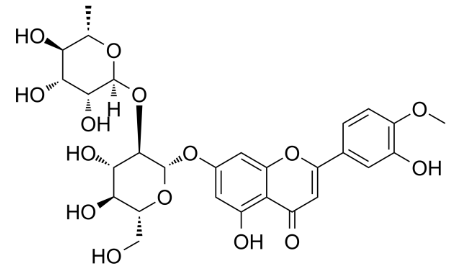 |
| Alnustone | 0.584653 | 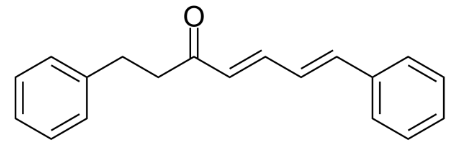 |
| Scopolamine | -0.15413 | **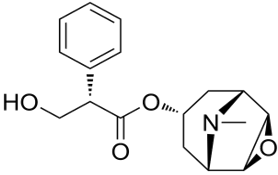** |
| 5,6,7-Trimethoxyflavone | 0.907863 | 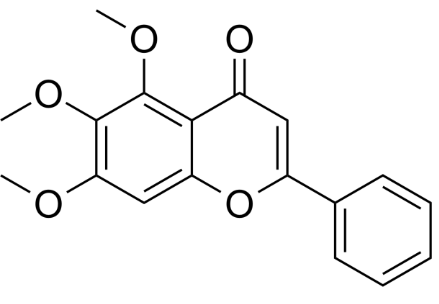 |
| Orcinol | 0.867645 | 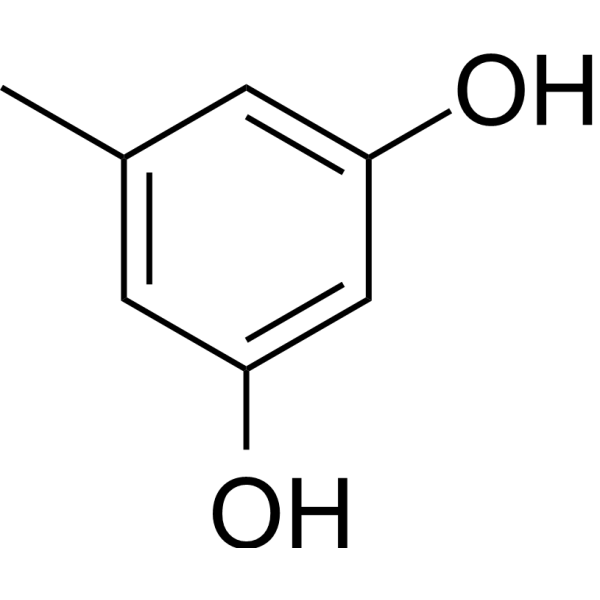 |
| 6-Hydroxy-4-methylcoumarin | -0.22796 | **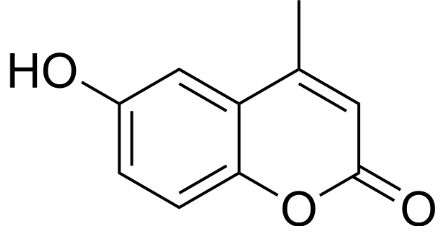** |
| 7-Hydroxy-4-methyl-8-nitrocoumarin | 0.771886 | **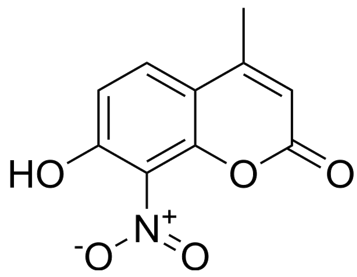** |
| (+)-Dipentene | 0.601816 | 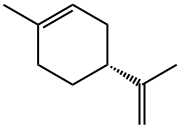 |
| Ethyl Coumarin-3-carboxylate | 0.5977 | 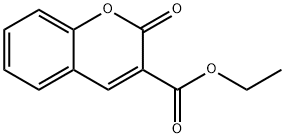 |

**Table S1**

Phytochemicals used in the primary screening (continued)

| **Phytochemicals** | **ε̃-value** | **Structure** |
| --- | --- | --- |
| Ethyl vanillin acetate | -0.55967 | 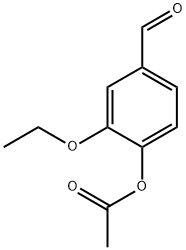 |
| Octyl gallate | 0.702374 | 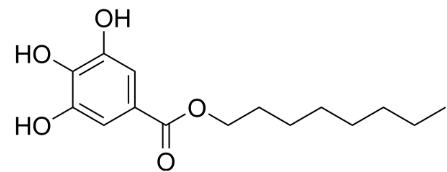 |
| S-(-)-Cotinine | 0.51287 | 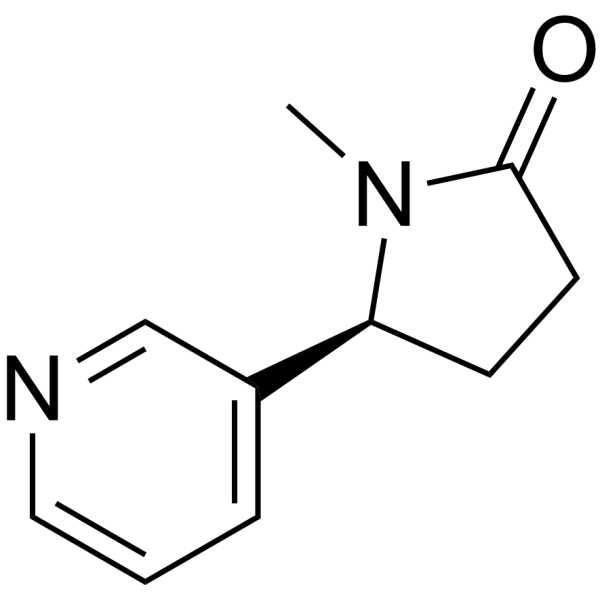 |
| Bz-RS-ISer(3-Ph)-Ome | 0.596776 | 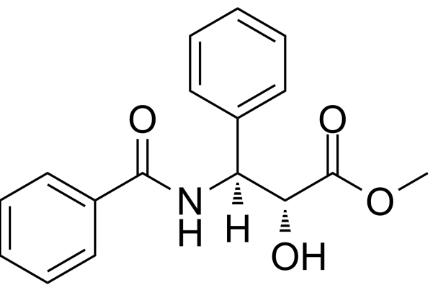 |
| 3,4-Dimethoxycinnamic acid | 0.845707 | 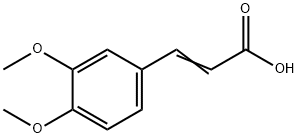 |
| 1,4-Naphthoquinone | 0.274022 | **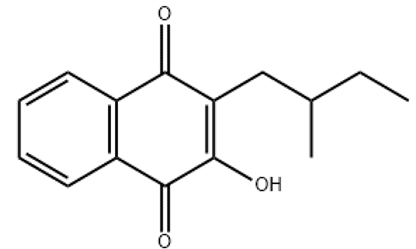** |
| Sequoyitol | 0.875827 | 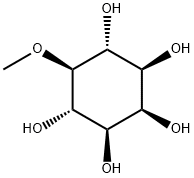 |
| 1-Indanone | 0.962028 | 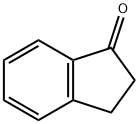 |
| Allitol | 0.798071 | 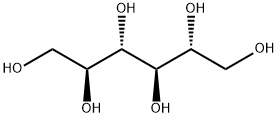 |
| D-(+)-Trehalose Anhydrous | 0.796431 | **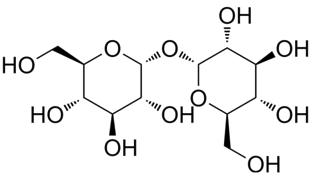** |
| D-(+)-Raffinose pentahydrate | 0.790547 | **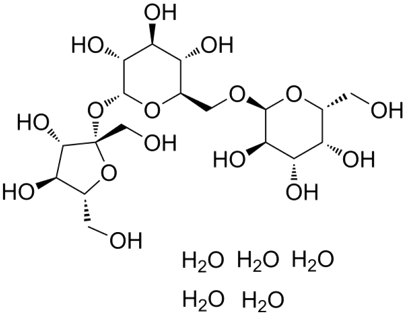** |
| Ethyl 4-Methoxycinnamate | 0.750904 | 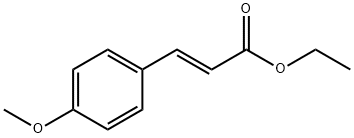 |

**Table S1**

Phytochemicals used in the primary screening (continued)

| **Phytochemicals** | **ε̃-value** | **Structure** |
| --- | --- | --- |
| 2'-Deoxyadenosine monohydrate | 0.716101 | 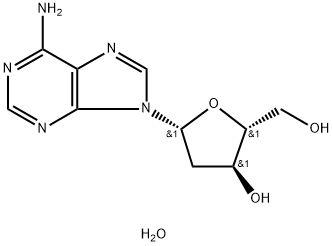 |
| Homogentisic Acid | 0.83649 | 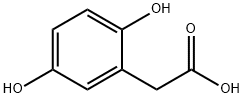 |
| Oxalic acid | 0.866762 | 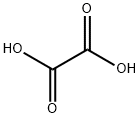 |
| Phenylacetaldehyde | 0.800293 | 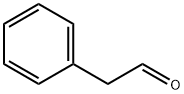 |
| Anthraquinone-2-carboxylic Acid | 0.840929 | 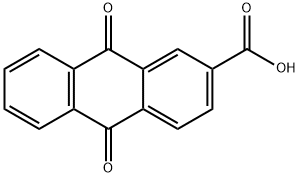 |
| 4-Hydroxyquinazoline | 0.801536 | 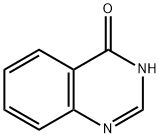 |
| Coumarin-3-carboxylic acid | 0.7505 | 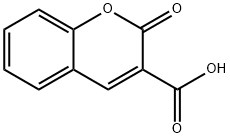 |
| 1-Naphthaleneacetic acid | -0.24224 | 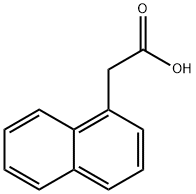 |
| Coumarin 151 | 0.828634 | 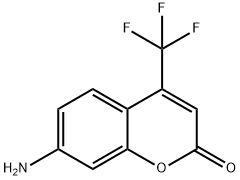 |
| 6-Hydroxycoumarin | 0.835313 | 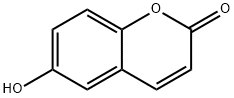 |
| Baicalein | -0.90429 | 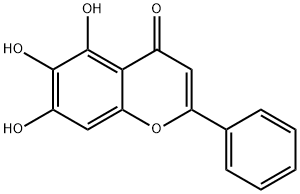 |
| ADP | 0.86613 | 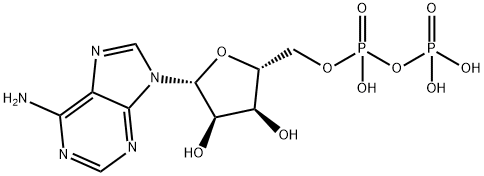 |

**Table S1**

Phytochemicals used in the primary screening (continued)

| **Phytochemicals** | **ε̃-value** | **Structure** |
| --- | --- | --- |
| Xanthosine Dihydrate | 0.813061 | 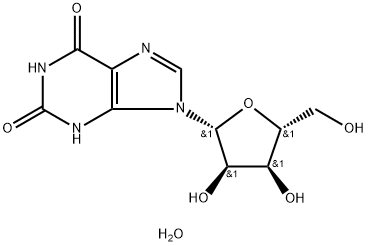 |
| Ethyl palmitate | 0.60463 | 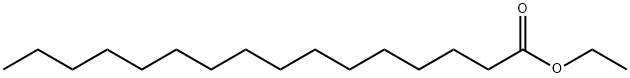 |
| Neryl acetate | -0.30802 | 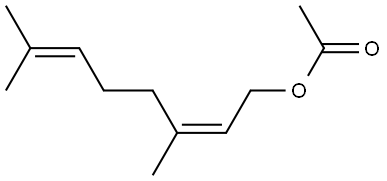 |
| 2',5'-Dihydroxyacetophenone | 0.579168 | 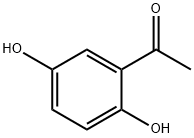 |
| 2'-Hydroxyacetophenone | 0.826045 | 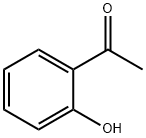 |
| Benzylacetone | 0.952276 | 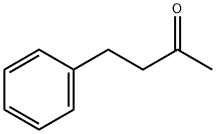 |
| Eugenyl acetate | 0.84577 | 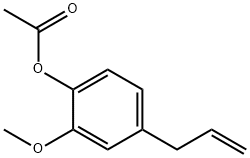 |
| 4',5-Dihydroxyflavone | 0.776384 | 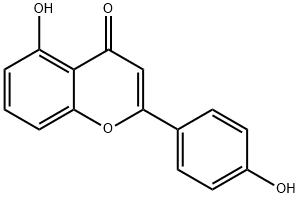 |
| Dihydrocoumarin | 0.707277 | 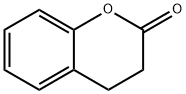 |
| 5,7-Dihydroxy-4-methylcoumarin | 0.456087 | 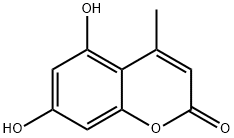 |
| 5-Methyl furfural | 0.585919 | 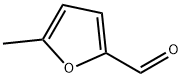 |
| Thymine | 0.444951 | 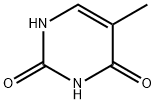 |
| Methyl palmitate | -0.31617 | 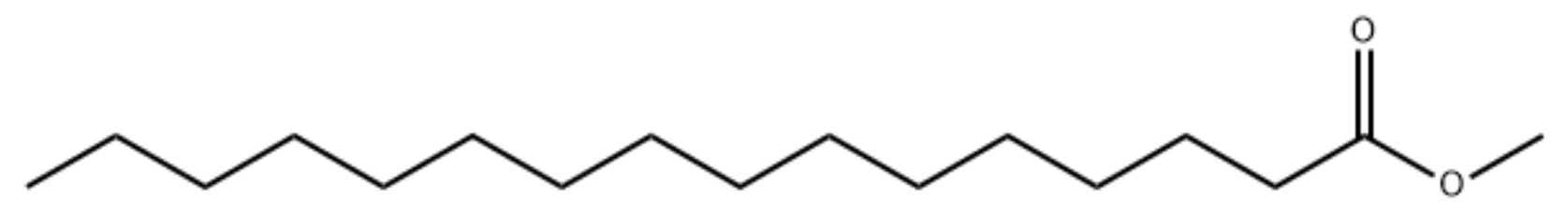 |

**Table S1**

Phytochemicals used in the primary screening (continued)

| **Phytochemicals** | **ε̃-value** | **Structure** |
| --- | --- | --- |
| Sophocarpine Monohydrate | 0.917731 | 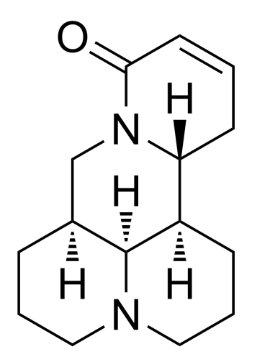 |
| Ligustilide | 0.77178 | 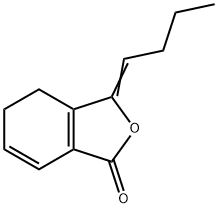 |
| Polygalacic acid | 0.725133 | 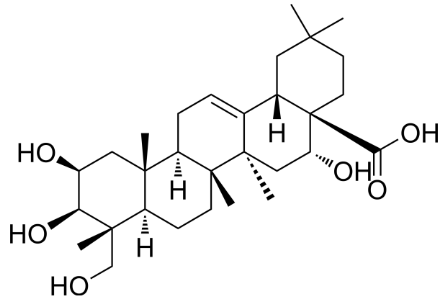 |
| Maackiain | 0.773494 | 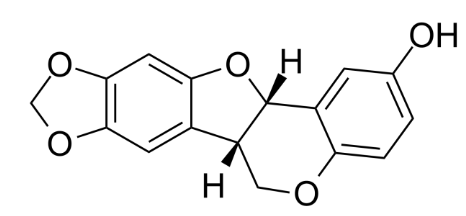 |
| (+)-Praeruptorin A | 0.729319 | 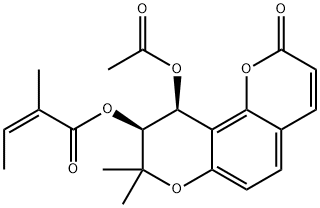 |
| Phorbol | 0.74076 | 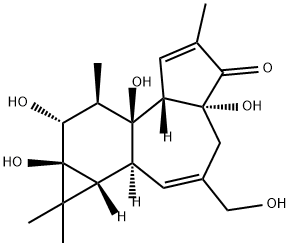 |
| Praeruptorin B | 0.575469 | 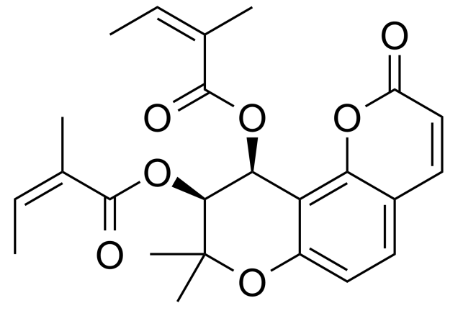 |
| (4S)-4-Hydroxy-L-isoleucine | 0.620335 | 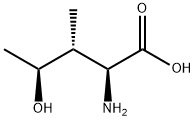 |
| N-Benzoyl-(2R,3S)-3-phenylisoserine | 0.634183 | 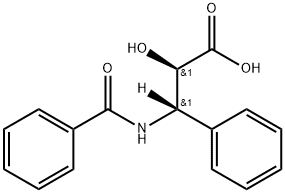 |

**Table S1**

Phytochemicals used in the primary screening (continued)

| **Phytochemicals** | **ε̃-value** | **Structure** |
| --- | --- | --- |
| Geraniin | -0.609977 | 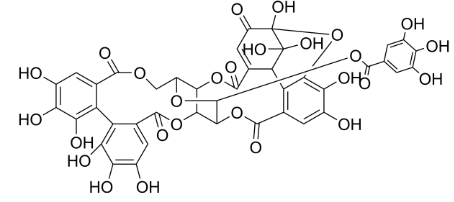 |
| Alliin | 0.774162 | 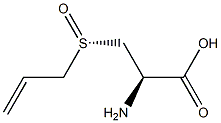 |
| Crassicauline A | 0.812066 | 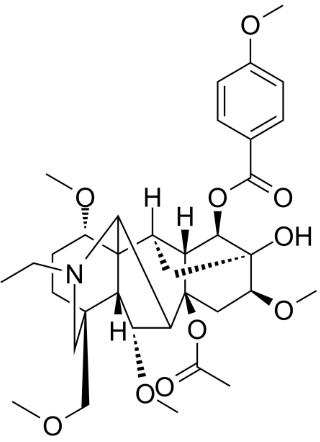 |
| 1,2,3,4,6-O-Pentagalloylglucose | 0.780442 | 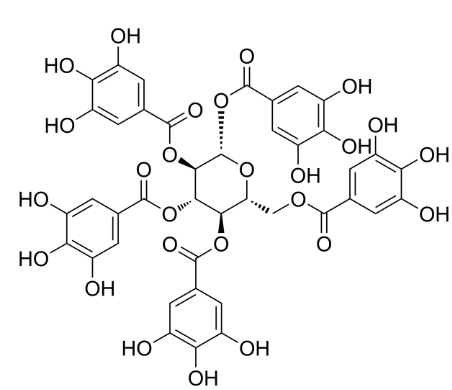 |

**Table S2**

Re-sensitization of colistin to bacteria in the host-mimicking conditions by BAI against different Gram-negative bacterial strains

| **Organism** | **Strain** | **Resistance determinants** | **Source** | MICs within CAMH | MIC within LPM | | |  |
| --- | --- | --- | --- | --- | --- | --- | --- | --- |
|  |  |  |  | CS | CS | CS+BAI (25 μg mL^-1^) | FC | |
| *Salmonella* Typhimurium | ATCC 14028 | Null | Lab stock | 1 | 32 | 1 | 32 | |
|  | 15E341 | MCR-1 | Lab stock[1] | 4 | 32 | 1 | 32 | |
|  | 15E164 | MCR-1 | Lab stock[1] | 4 | 64 | 1 | 64 | |
|  | 15E193 | MCR-1 | Lab stock[1] | 8 | 64 | 1 | 64 | |
|  | Δ*pmrA* | Null | Lab stock[2] | 1 | 2 | 0.5 | 4 | |
|  | Δ*pmrB* | Null | Lab stock[2] | 1 | 2 | 0.5 | 4 | |
|  | Δ*phoP* | Null | Lab stock[2] | 1 | 2 | 0.5 | 4 | |
|  | Δ*phoQ* | Null | Lab stock[2] | 1 | 2 | 0.5 | 4 | |
|  | Δ*eptA* | Null | Lab stock[2] | 1 | 2 | 0.5 | 4 | |
|  | Δ*arnT* | Null | Lab stock[2] | 1 | 4 | 0.5 | 8 | |
| *Escherichia coli* | ATCC 25922 | Null | Lab stock | 2 | 64 | 2 | 32 | |
|  | MM41-1 | MCR-1 | Lab stock[3] | 4 | 32 | 2 | 16 | |
|  | WF94 | MCR-1 | Lab stock[3] | 8 | 32 | 2 | 16 | |
|  | WFW2 | MCR-1 | Lab stock[3] | 8 | 64 | 1 | 64 | |
| *Klebsiella pneumoniae* | ATCC 700603 | Null | Lab stock | 2 | 64 | 1 | 64 | |
|  | 2587CR156 | MCR-1 | Lab stock[2] | 8 | 64 | 2 | 32 | |
|  | ZJ18-19 | *mgrB*-disrupted | Lab stock[2] | 4 | 64 | 2 | 32 | |
|  | CMG | *mgrB*-disrupted | Lab stock[2] | 8 | 16 | 1 | 16 | |

**Table S3**

Oligonucleotide primers used in the current study

| **Primers** | **Sequence (5’-3’)** |
| --- | --- |
| Q-pmrA-F | TGCTGGATTTAGGGCTGC |
| Q-pmrA-R | GCCAACCGTCAGTTCAC |
| Q-pmrB-F | CCTTCGCCAGCGTTTAAT |
| Q-pmrB-R | TTTGCAGTTCGGCGAGC |
| pmrA-HA-F | TTGGGCTAGCAGGAGGAATTATGAAGATACTGATTGTTGAAGACG |
| pmrA-HA-R | GGCAATTCCGACGTCGAATTTTAAGCGTAATCTGGAACATCGTATGGGTAGCTTTCCTCAGTGGCAACC |
| ParnT- F | AATCTGCTCTGATGCCGCATAGGATCGCACCGCTCGG |
| ParnT-R | TGAATGAAATTTTTTTAGTCATCATTTTTCCTTCAGCCATTGAAAG |
| PeptA-F | GCATAGTTAAGCCAGCCCCGAGTGGCTGGGTTGCTT |
| PeptA-R | GCCGTTAATAATGAATGAAATTTTTTTAGTCATGTTGATGCGTCCATCGATTC |
| Delete-pmrB-F | GGTTCGCGGGTTTGGCTACATGCTGGTTGCCACTGAGGAAAGCTAAGTGAATTGCAGCATTACACGTCTTGAG |
| Delete-pmrB-R | TGCTGATTGTCAGCAGTTTTATCTATGTGTGGGTCACGACGTATTAAACGGGCTGACATGGGAATTAGCCA |
| Delete-pmrA-F | GCCGCAGATGATATTCTGCAACCGTGCAGGAGACTAAGCGAATGATGCAGCATTACACGTCTTGAG |
| Delete-pmrA-R | CTGGCGAAGGGTCATCGCTCTTCGCTGAAAACGCATCAGGCTCACGCTGACATGGGAATTAGCCA |
| RT-16S-F | AGATACCCTGGTAGTCCACGC |
| RT-16S-R | TTGCGGGACTTAACCCAAC |

**Table S4**

Plasmid constructs used in the current study

| **Plasmids** | **Genotype or phenotype** | **References** |
| --- | --- | --- |
| pBAD24::*pmrA*-HA | pBAD24 carrying *pmrA* with the HA tag | Lab stock[2] |
| P*_arnT_*-Lux | The promoter of *arnT* was fused with LuxCDABE | Lab stock[2] |
| P*_eptA_*-Lux | The promoter of *eptA* was fused with LuxCDABE | Lab stock[2] |

Note: The plasmid pBAD24 vector was utilized as the backbone. An HA tag was fused to the *pmrA* fragment, and this fusion construct was inserted into pBAD24 to produce the recombinant plasmid pBAD24::*pmrA*-HA. Using an identical cloning strategy, the promoters of *arnT* and *eptA* genes, as well as the bacterial luciferase operon LuxCDABE, were separately fused and cloned into the pUC19 backbone to generate reporter plasmids P*_arnT_*-Lux and P*_eptA_*-Lux, respectively, which were used to monitor the expressions of these genes *in vivo*.

**SI References**

[1] Sun RY, Fang LX, Ke BX, *et al.* Carriage and Transmission of *mcr*-1 in *Salmonella* Typhimurium and Its Monophasic 1,4,[5],12:i:- Variants from Diarrheal Outpatients: a 10-Year Genomic Epidemiology in Guangdong, Southern China. Microbiol Spectr. 2023; 11(1):e0311922. doi:10.1128/spectrum.03119-22

[2] Zhong ZX, Zhou S, Liang YJ, *et al.* Natural flavonoids disrupt bacterial iron homeostasis to potentiate colistin efficacy. Sci Adv. 2023; 9(23):eadg4205. doi:10.1126/sciadv.adg4205

[3] Liu KD, Jin WJ, Li RB, *et al.* Prevalence and molecular characteristics of *mcr*-1-positive *Escherichia coli* isolated from duck farms and the surrounding environments in coastal China. Microbiol Res. 2023; 270:127348. doi:10.1016/j.micres.2023.127348
